# ICAM-1 promotes cancer progression by regulating SRC activity as an adapter protein in colorectal cancer

**DOI:** 10.1038/s41419-022-04862-1

**Published:** 2022-04-29

**Authors:** Eun-Ji Lim, Jae-Hyeok Kang, Yeon-Ju Kim, Seungmo Kim, Su-Jae Lee

**Affiliations:** Department of Life Science, Research Institute for Natural Sciences, Hanyang University, Seoul, 04763 Korea

**Keywords:** Metastasis, Oncogenes

## Abstract

Colorectal cancer (CRC) has a 5-year survival rate of <10%, as it can metastasize to the lungs and liver. Anticancer drugs and targeted therapies used to treat metastatic colorectal cancer have insufficient therapeutic efficacy and are associated with complications. Therefore, research to develop new targeted therapeutics is necessary. Here, we present a novel discovery that intracellular adhesion molecule-1 (ICAM-1) is a potential therapeutic target to enhance therapeutic effectiveness for CRC. ICAM-1 is an important regulator of cell–cell interactions and recent studies have shown that it promotes malignancy in several carcinomas. However, little is known about its effect on CRC. Therefore, we conducted a study to define the mechanism by which ICAM-1 acts. ICAM-1 is phosphorylated by tyrosine-protein kinase Met (c-MET), and phosphorylated ICAM-1 can interact with SRC to increase SRC activity. Consequently, ICAM-1 may further accelerate SRC signaling, promoting the malignant potential of cancer. In addition, treatment with antibodies targeting ICAM-1 showed excellent therapeutic effects in reducing metastasis and angiogenesis. These findings suggest for the first time that ICAM-1 is an important adapter protein capable of mediating the c-MET-SRC signaling axis. Therefore, ICAM-1 can be used as a novel therapeutic target and a metastatic marker for CRC.

## Introduction

Colorectal cancer (CRC) is one of the three most common cancers, and its incidence is rapidly increasing worldwide because of aging society and increased consumption of westernized diets. CRC is divided into four stages, and during stage 4 it metastasizes to the lungs and liver with a 5-year survival rate of <10% [1]. Therefore, prevention of metastasis is a very important factor in increasing the survival rate of patients. Various anticancer drugs, such as 5-FU, oxaliplatin, and irinotecan, that are used to treat metastatic CRC have displayed side effects, such as mucositis, suppression of bone marrow function, and dehydration. To address this problem, targeted therapeutics are being developed. However, targeted therapy for CRC using bevacizumab (VEGF inhibitor), cetuximab (EGFR inhibitor), and other drugs, has exhibited complications or is ineffective. Therefore, the development of new targeted therapeutics is urgently needed [2–4].

It is known that the expression of immunoglobulin in CRC is much higher than that of normal tissue, and it is highly likely to be used as a new therapeutic target [5, 6]. Intracellular adhesion molecule (ICAM) belongs to the immunoglobulin superfamily. The members of this family have two or more extracellular immunoglobulin-like domains and a cytoplasmic tail containing tyrosine (Tyr) with a signaling function [7]. ICAM-1 is an important transmembrane protein that stabilizes cell–cell interactions and promotes leukocyte–endothelial migration [8]. It is highly expressed in endothelial cells, peripheral lymphoid tissues, and some parenchymal cells. However, it is also upregulated in various cancers, such as melanoma, breast cancer, and lymphoma [9]. ICAM-1 primarily acts as an adhesive molecule; however, it can also promote metastasis and angiogenesis and weaken the immune response in cancer cells. Therefore, it is known as a biomarker in various cancer types, but its function is not well known in CRC [7, 10, 11].

SRC is a representative signaling molecule that regulates metastasis and angiogenesis. Moreover, it shows high activity in various cancers, such as liver, lung, breast, and pancreatic cancers. In particular, SRC expression is five to eight times higher in premalignant colorectal polyps than in normal mucosa. It also influences the survival of CRC patients. Therefore, inhibition of SRC may have an important therapeutic effect in CRC, and many studies are currently underway [12–14].

Previously, we studied the role of ICAM-1 in various cancers[15, 16]; however, its role and mechanism in CRC has not been fully elucidated. Therefore, in this study, the role and mechanism of ICAM-1 were investigated in order to present it as a therapeutic target for CRC. We propose that phosphorylated ICAM-1 as an adapter protein may modulate cancer malignancy by further promoting the activity of SRC on the c-MET-SRC axis.

## Results

### ICAM-1 promotes poor prognosis in CRC by regulating epithelial–mesenchymal transition (EMT) and angiogenesis

**Fig. 1:**
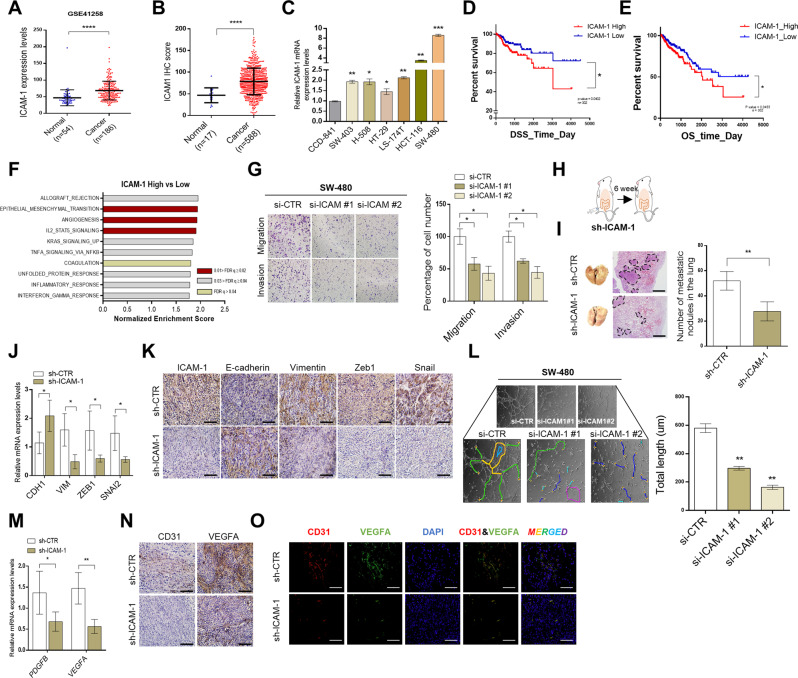
ICAM-1 promotes poor prognosis by regulating EMT and angiogenesis in colorectal cancer. **A** ICAM-1 expression data in normal colon tissue (*n* = 54) and colorectal cancer tissue (*n* = 186) was obtained from the Gene Expression Omnibus (GEO) database. **B** Tissue microarray analysis of ICAM-1 expression in normal (*n* = 17) and cancer (*n* = 588) type of colorectal tissues. **C** qRT-PCR was performed to detect the expression of ICAM-1 in various colorectal cancer cell lines and normal colon cell. **D**, **E** Kaplan–Meier survival analysis cohorts based on low and high expression of ICAM-1 in colorectal cancer. (TCGA, *n* = 302). **F** GSEA (GSE4183) analysis was performed in colorectal cancer that ICAM-1 high expressed for hallmarks of cancer progression. NES normalized enrichment score; FDR q val false discovery rate *q*-value. **G** Transwell chamber assays were performed to determine whether expression of ICAM-1 modulates EMT after silencing ICAM-1 using SW-480 cells. **H** SW-480 cells transfected with sh-CTR and sh-ICAM-1 (3 × 10^6^) were injected into the cecal wall of athymic BALB/c-nude (8-weeks old) mice (*n* = 4). **I** Representative H&E staining images of lung metastases. And the graph shows the number of lung metastatic lesions in each mouse**. J**, **K** qRT-PCR and IHC staining of expression levels for EMT markers and regulators in tumor tissues of sh-CTR and sh-ICAM-1 injected mice. **L** Tube-forming ability of HUVEC cells was performed by incubation with SW-480 in which ICAM-1 was silenced. Tube formation was assessed after 2 h using light microscopy, and the Image J program was used to analyze tube length. **M**, **N** qRT-PCR and IHC staining of expression levels for that angiogenesis-related markers and growth factors in tumor tissues of sh-Ctr and sh-ICAM-1 injected mice. **O** Representative immunofluorescence image for CD31 (red) and VEGFA (green) in mouse tumors. Scale bar, 100 μm, β-actin and positive control were used as a control for normalization. Data are presented as mean ± SD and analyzed by Student’s *t*-tests. *P < 0.05; **P < 0.01; ***P < 0.001.

To determine whether ICAM-1 is highly expressed as an oncogene in CRC patients, we analyzed ICAM-1 expression using the gene expression omnibus (GEO) online database and colon cancer tissue microarray. Consequently, we found that ICAM-1 expression was higher in CRC tissues than in normal tissues, and analysis using cell lines also confirmed that ICAM-1 expression was higher in malignant cells than in normal cells (Fig. 1A–C). Kaplan–Meier survival analysis showed a positive correlation between ICAM-1 expression and poor disease-specific and overall survival (Fig. 1D, E). These results suggest that ICAM-1 is highly expressed in patients with CRC and may influence their survival.

To explore the biological function of ICAM-1 in CRC, we first screened its correlation with cancer hallmarks using the GSEA database. We found that ICAM-1 was positively correlated with a set of signature genes involved in EMT and angiogenesis (Fig. 1F). Therefore, we measured the migration/invasion ability of cancer cells according to ICAM-1 expression using either Matrigel-uncoated or Matrigel-coated transwell chamber. As a result, the migration and invasion of SW-480 cells transfected with si-ICAM-1 were significantly inhibited compared to the control group (Fig. 1G). In addition, the expression of EMT markers and regulators such as N-Cadherin (N-CAD), Vimentin (VIM), Snail, and Slug was also reduced, as assessed by quantitative reverse-transcriptase polymerase chain reaction (qRT-PCR) and western blotting (Supplementary Fig. S1A, B). Conversely, when ICAM-1 was overexpressed in HT-29 cells, increased cell migration and invasion, and upregulated expression of EMT markers and regulators were observed (Supplementary Fig. S1C, E, F). We further investigated the effect of ICAM-1 knockdown on metastasis by creating an orthotopic mouse model in which SW-480 cells were injected into the cecal wall (Fig. 1H). We showed by hematoxylin and eosin (H&E) staining, qRT-PCR and immunohistochemistry (IHC) experiments that reduction in ICAM-1 expression inhibited the formation of lung metastatic foci and the expression of EMT-related markers and transcription factors (Fig. 1I–K and Supplementary Fig. S1J, K). The mouse group injected with sh-ICAM-1 into the tail vein had a reduced number of liver and lung metastatic lesions compared to that of the sh-Ctr group (Supplementary Fig. S1G–1I).

Next, since ICAM-1 has a positive correlation with angiogenesis as well as EMT, tube formation analysis was performed using human umbilical vein endothelial cells (Fig. 1F). Reduction of ICAM-1 expression in SW-480 cells downregulated the angiogenic capacity of the cells and the expression level of angiogenic regulators such as VEGFA, PDGF-BB, and FGF2 (Supplementary Fig. S1A, B and Fig. 1L). However, contradictory results were observed in the ICAM-1-overexpressed HT-29 vein cells (Supplementary Fig. S1D–1F). Furthermore, the results of qRT-PCR, IHC, and immunofluorescence (IF) staining demonstrated a reduction in the levels of angiogenesis-related markers and growth factors with downregulation of ICAM-1, in the orthotopic mouse model system (Fig. 1M–O and Supplementary Fig. S1J, K). These results strongly indicate that ICAM-1 is associated with the poor prognosis of CRC, and it exerts its effects by regulating metastasis and angiogenesis.

### ICAM-1 regulates malignancy potential through SRC-STAT3 signaling

**Fig. 2:**
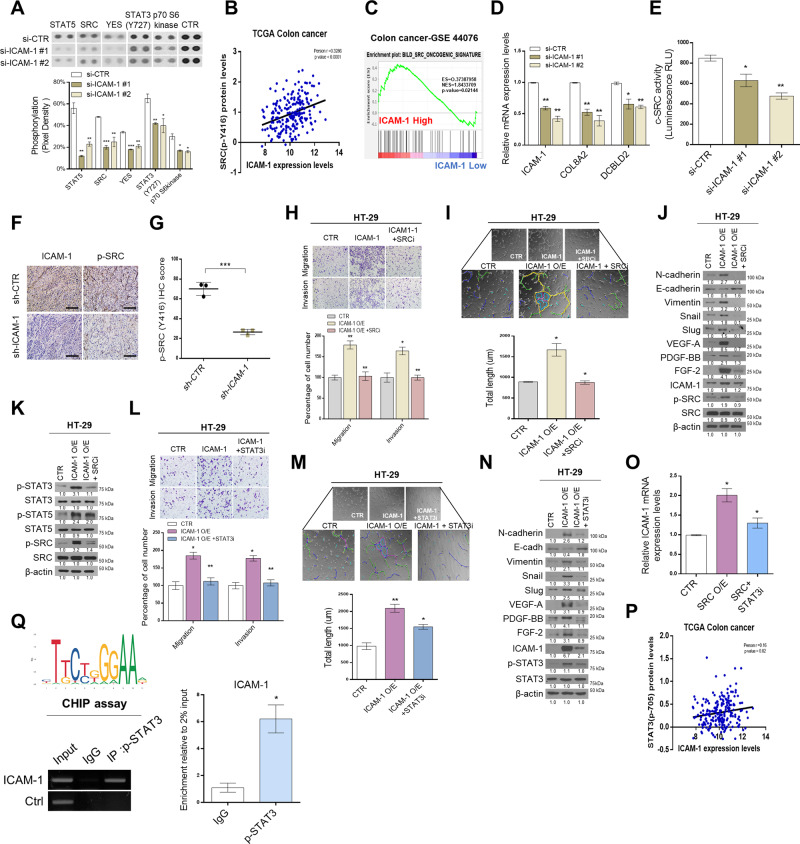
ICAM-1 regulates malignancy potential through SRC-STAT3 signaling. **A** Kinase assay was performed on SW-480 cells in which ICAM-1 was silenced, and the intensity of each dot was analyzed using the Image J program (*n* = 2). **B** Pearson correlation analysis of ICAM-1 and p-SRC expression levels in TCGA colon cancer patient cohort. **C** GSEA of SRC oncogene signature gene to ICAM-1 expression in colorectal cancer patients from GSE44076. **D** qRT-PCR of representative SRC signature genes was performed in SW-480 cells transfected with si-CTR and si-ICAM-1. **E** SRC kinase assay was detected using luminescence in SW-480 cells with reduced ICAM-1 expression. **F**, **G** IHC staining assays and staining intensity score analysis of p-SRC in control and ICAM-1 knockdown mouse tissues. **H**, **I** Transwell chamber assay and tube formation assay were performed using HT-29 cells with overexpressed ICAM-1 and SRC inhibitors treatment. Tube formation was assessed after 2 h using light microscopy, and the Image J program was used to analyze tube length. **J** Western blot on the expression level of EMT and angiogenesis-related regulators in HT-29 cells. **K** Western blotting in HT-29 cells after treatment with overexpressed ICAM-1 and SRC inhibitors. **L**–**N** Transwell chamber assay, tube formation assay, and western blot were performed using HT-29 cells with overexpressed ICAM-1 and STAT3 inhibitors treatment. Tube formation was assessed after 2 h using light microscopy, and the Image J program was used to analyze tube length. **O** Quantitative real-time PCR analysis of ICAM-1 mRNA expression in HT-29 after treatment with overexpressed SRC and STAT3 inhibitors treatment. **P** Pearson correlation analysis of ICAM-1 and p-STAT3 expression levels in TCGA colon cancer patient cohort. **Q** The ChIP assay showed that p-STAT3 directly binds to the ICAM-1 promoters at specific sites in SW-480 cells. β-actin and positive control were used as a control for normalization. Data are presented as mean ± SD and analyzed by Student’s *t*-tests. *P < 0.05; **P < 0.01; ***P < 0.001.

To determine the signaling molecules directly regulated by ICAM-1, we performed kinase arrays. As a result, the top five candidates that were the most downregulated when ICAM-1 knocked down in SW-480 cells were as follows: STAT5, SRC, YES, STAT3 (Y727), and p70 S6 kinase (Fig. 2A and Supplementary Fig. S2A). Among them, phosphorylation of SRC and p70 S6 kinase was most significantly inhibited except for candidates acting as transcription factors. Next, we analyzed the Cancer Genome Atlas (TCGA) database to determine whether the expression level of ICAM-1 is correlated with kinase activity. We found that only p-SRC was positively correlated with ICAM-1 expression in CRC patients (Fig. 2B and Supplementary Fig. S2B). Moreover, we directly confirmed that SRC phosphorylation is affected by knockdown or overexpression of ICAM-1 (Supplementary Fig. S2C). Furthermore, GSEA analysis (GSE44076 dataset) revealed a positive correlation between ICAM-1 and the SRC oncogenic signature gene (Fig. 2C). The expression of the SRC oncogenic signature target gene decreased in ICAM-1-silenced SW-480 cells, as revealed by qRT-PCR analysis (Fig. 2D). Additionally, an in vitro SRC kinase assay revealed reduced c-SRC activity in ICAM-1 knocked down cells (Fig. 2E). The IHC staining score of p-SRC was also significantly attenuated in the sh-ICAM-1 xenograft group (Fig. 2F, G). From these results, we found that ICAM-1 regulates SRC phosphorylation in CRC. Previous studies have reported that SRC plays an important role in regulating EMT and angiogenesis [17]. Therefore, we evaluated the ability of SRC to affect EMT and angiogenesis in CRC using a transwell cell migration/invasion assay and tube formation assay. As a result, metastasis and angiogenesis were regulated according to SRC expression level (Supplementary Fig. S2D–I). Additionally, we performed rescue experiments to determine whether the aggressive abilities of ICAM-1 are mitigated by inhibition of SRC activity. Interestingly, the increased migratory/invasive, and tube formation abilities by ICAM-1 were both decreased along with the expression levels of EMT and angiogenesis-related factors when ICAM-1-transfected HT-29 cells were treated with PP2 (SRC inhibitor) (Fig. 2H–J). In parallel with this result, our data showed that their abilities were similarly rescued when the expression of SRC was interfered (Supplementary Fig. S2J–L). Previous studies have known that SRC can downstream regulate PI3K/RAS/STAT signaling [18–20]. Similarly, transcription factors such as STAT3 and STAT5 were also observed to be regulated in our kinase array results (Fig. 2A). Therefore, to identify downstream player of the ICAM-1/SRC axis, we compared the phosphorylation levels of STAT3 and STAT5 by overexpressing ICAM-1 and treating with an SRC inhibitor and found that p-STAT3 was strongly downregulated (Fig. 2K). Furthermore, we performed transwell cell migration/ invasion assay and tube formation assays to determine whether the malignancy potential induced by ICAM-1 was mitigated by inhibiting STAT3 activity. The migration, invasion, and tube formation abilities of the cells diminished and expression of the EMT and angiogenesis associated factors also reduced when ICAM-1-overexpressed HT-29 cells were treated with WP1066 (STAT3 inhibitor) (Fig. 2L–N). Taken together, the ICAM-1/SRC/STAT3 axis affects the regulation of EMT and angiogenesis. In the above experiments, we also observed that the expression level of ICAM-1 decreased when SRC and STAT3 activities were inhibited (Fig. 2J, N). Therefore, to determine whether SRC/STAT3 signaling could regulate ICAM-1 expression, we overexpressed SRC and then treated the STAT3 inhibitor. When the STAT3 inhibitor was treated, the expression of ICAM-1 was significantly reduced, and it was also found that there was a positive correlation between the expression levels of ICAM-1 and p-STAT3 in the TCGA database (Fig. 2O, P and Supplementary Fig. S2M). Since STAT3 is known to directly regulate ICAM-1 expression in various cancer types, chromosome immunocompatibility (CHIP) assay was performed to investigate this role of STAT3 in CRC [21–23]. We observed that STAT3 regulated ICAM-1 expression by binding to the promoter of ICAM-1 (Fig. 2Q). Additionally, the expression level of ICAM-1 was decreased in STAT3 knockdown cells (Supplementary Fig. S2N).

In conclusion, we demonstrated that EMT and angiogenesis are regulated by the ICAM-1/SRC/STAT3 axis in CRC. Moreover, SRC and STAT3, which are activated as ICAM-1 level increases, again affect ICAM-1 expression, resulting in the formation of a positive feedback loop.

### The Tyr512 residue of ICAM-1 is as an important cofactor of SRC activity

**Fig. 3:**
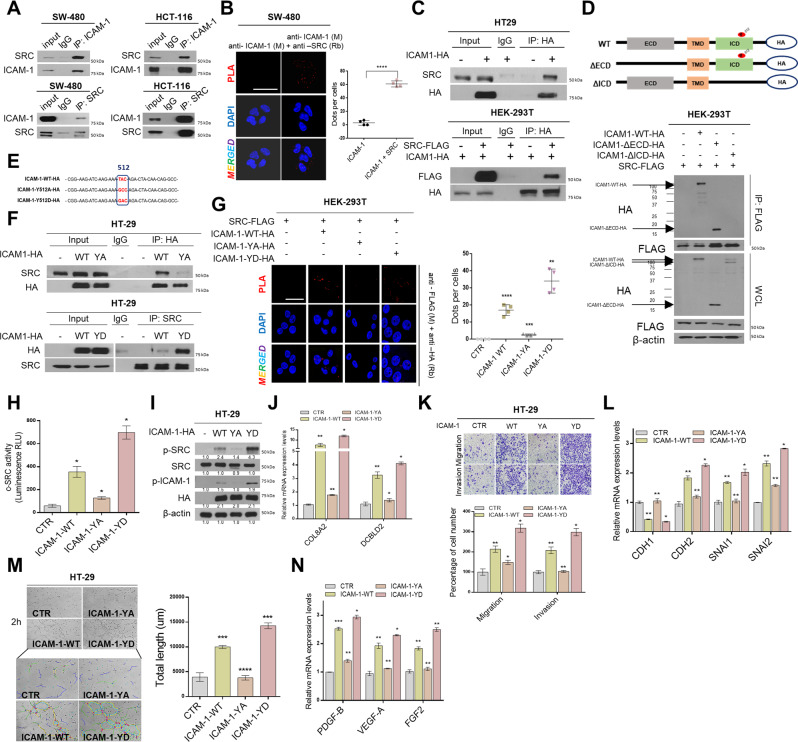
The Tyr512 residue of ICAM-1 serves as an important cofactor in SRC signaling activity. **A** Co-IP was performed. The cell lysate was immunoprecipitated with an ICAM-1 and SRC antibody or an immunoglobulin G (lgG) to detect the protein interaction between ICAM-1 and SRC in SW-480 and HCT-116 cells. **B** Representative confocal images of in situ PLA staining in SW-480 cells using anti-ICAM-1 (M) and anti-SRC (Rb). The graph shows the number of dots per cell counted using ImageJ software. Scale bar = 100 μm. **C** Co-IP analysis shows protein interaction of ICAM-1 with SRC using transfected HT-29 and HEK293T cells. **D** Co-IP assay is showed that SRC binds to the intracellular domain of ICAM-1. HEK293T cells were co-transfected with expression plasmids encoding FLAG–SRC and deletion construct of HA-ICAM-1. **E** Schematic diagram of the point mutation of Tyr residue in ICAM-1. **F** Co-IP experiment between SRC and point mutation structure of ICAM-1. HEK293T cells were co-transfected with expression plasmids encoding FLAG–SRC and point mutation construct of HA-ICAM-1. WT, wild-type ICAM-1; YA, ICAM-1 Y512A mutant (inactivation form); YD, ICAM-1 Y512D (activation form). **G** Representative confocal images of in situ PLA staining in HEK293T cells using anti-FLAG (M) and anti-HA (Rb). The graph shows the number of dots per cell counted using ImageJ software. Scale bar = 100 μm. **H**, **I** p-SRC activity was detected using Src kinase assay and Western blot in HT-29 cells transfected with various ICAM-1 point mutation constructs. **J** qRT-PCR of representative SRC signature genes was performed in HT-29 cells. **K** Migration/Invasion assays of ICAM-1 point mutation constructs in HT-29 cells. **L** qRT-PCR for marker and transcription factor of EMT were performed. **M** Tube formation assay of ICAM-1 point mutation constructs in HT-29 cells. Tube formation was assessed after 2 h using light microscopy, and the Image J program was used to analyze tube length. **N** qRT-PCR for angiogenic factors were performed. β-actin and positive control were used as a control for normalization. Data are presented as mean ± SD and analyzed by Student’s *t*-tests. *P < 0.05; **P < 0.01; ***P < 0.001.

To investigate the mechanism by which ICAM-1 activates SRC, we first determined whether they bind to each other. Through co-immunoprecipitation (co-IP) and Proximity ligation assay (PLA) experiments, we confirmed that ICAM-1 and SRC were bound in the SW-480 and HCT-116 cell lines (Fig. 3A, B). Similar results were observed in ICAM-1 and SRC overexpressing-HT-29 and -HEK293T cell lines (Fig. 3C). Next, we created a truncated deletion construct of ICAM-1, consisting of an intracellular domain deletion (ΔICD) and an extracellular domain deletion (ΔECD). When ICAM-1-ΔICD and SRC were co-transfected into HEK293T cells, we observed through co-IP and PLA that ICAM-1 did not bind to SRC (Fig. 3D and Supplementary Fig. S3A). Therefore, we identified that the intracellular domain of ICAM-1 binds to the SRC. Recent studies have shown that Tyr residues in the intracellular domain of ICAM-1 can be important targets for novel anti-inflammatory agents in regulating various signaling pathways [24, 25]. However, the exact functional mechanism of Tyr residues in CRC cells has not been elucidated. Therefore, to evaluate whether the Tyr residue affects the binding of ICAM-1 to SRC, we created a phosphomimetic mutant in which the Tyr residue was either unphosphorylated or remains active without the need to be phosphorylated (Fig. 3E) [26]. Co-IP and PLA results revealed that when the Tyr residue was substituted with alanine, it did not bind to SRC, whereas when it was substituted with aspartic acid, the binding significantly increased (Fig. 3F, G and Supplementary Fig. S3B). The results of SRC kinase assay, western blotting, and qRT-PCR confirmed that the Tyr512 residue of ICAM-1 affected not only the binding of SRC but also the activity and expression of the SRC oncogenic signature target gene (Fig. 3H–J). Moreover, migration/invasiveness and angiogenesis, regulated by ICAM-1, increased in the group with phosphomimetic ICAM-1 form and significantly decreased in the group with inactivated ICAM-1 form substituted with alanine compared to that in the wild-type (Fig. 3K–N and Supplementary Fig. S3C). Collectively, these results showed that the Tyr512 residue of ICAM-1 can directly bind to SRC, thereby regulating SRC signaling activity.

### ICAM-1 as an important adapter protein in the c-MET–SRC axis

**Fig. 4:**
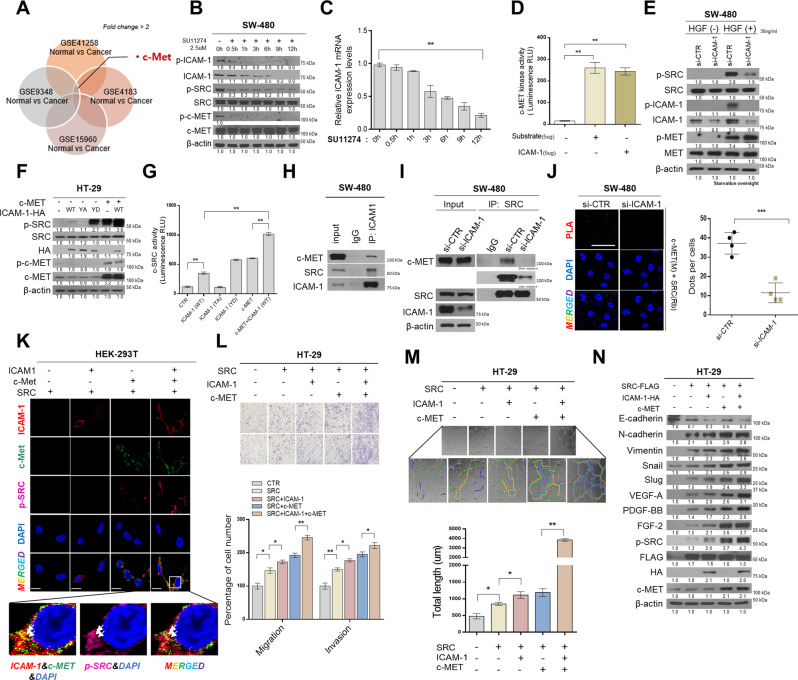
ICAM-1 plays a role as an important adapter protein in the c-MET–SRC axis. **A** RTK screening with high expression in colorectal cancer compared to normal using GSE (GSE41258, GSE9348, GSE4183, GSE15960) analysis. Fold change > 2. **B**, **C** Detection of phosphorylation and expression of ICAM-1 in SW-480 cells after SU11274 treatment (2.5 μmol/L, c-MET inhibitor). **D** c-MET kinase assay at the protein level. ICAM-1 was purified using an HA-tagged protein purification kit and added instead of ligand. **E** Western blot analysis was performed in ICAM-1 silenced SW-480 cells after 24 h treatment with HGF (30 ng/μL). **F** Western blot showed that the intensity of p-SRC was significantly increased when c-MET and ICAM-1 were co-expressed. **G** SRC kinase assay also showed that the activity of p-SRC was highly increased when c-MET and ICAM-1 were co-expressed in HT-29 cells. **H** co-IP assay was performed. The cell lysate was immunoprecipitated with an ICAM-1 antibody or lgG to detect the protein interaction between ICAM-1 and SRC or c-MET and ICAM-1 in SW-480 cells. **I** After silencing the expression of ICAM-1 in SW-480 cells, co-IP experiment between SRC and c-MET was performed. **J** Representative confocal images of in situ PLA staining with anti-c-MET (M) and anti-SRC (Rb) in SW-480 cells with suppressed ICAM-1 expression. The graph shows the number of dots per cell counted using ImageJ software. Scale bar = 100 μm. **K** Immunofluorescence analysis for p-c-MET, ICAM-1, and p-SRC protein levels in HEK293T cells overexpressing c-MET, ICAM-1, and SRC (magnification, × 200, scale bar, 50 μm). **L**–**N** Transwell chamber assay, tube formation assay, and western blot were performed using HT-29 cells with overexpressed c-MET, ICAM-1 and SRC. Tube formation was assessed after 2 h using light microscopy, and the Image J program was used to analyze tube length. Data are presented as mean ± SD and analyzed by Student’s *t*-tests. *P < 0.05; **P < 0.01; ***P < 0.001.

Next, we screened four GEO databases to determine which receptor tyrosine kinases (RTKs) can phosphorylate ICAM-1 to promote SRC binding. We discovered that c-MET was more expressed in CRC patients than in normal tissues (Fig. 4A). Thus, we confirmed that the phosphorylation of ICAM-1 was reduced by treatment with SU11274 (c-MET inhibitor) in a time-dependent manner (Fig. 4B). In addition, phosphorylation of SRC was also reduced by treatment with a c-MET inhibitor, as c-MET is known to regulate various signals such as SRC, RAS, and PI3K [27–29]. Furthermore, we found that the total expression of ICAM-1 decreased gradually when the c-MET inhibitor was administered, due to the positive feedback loop of ICAM-1/SRC/STAT3 (Fig. 4C). Next, a c-MET kinase assay was performed after purifying the ICAM-1 protein to identify whether c-MET can directly phosphorylate ICAM-1. We observed that kinase activity increased when purified ICAM-1 was added instead of the substrate (Fig. 4D). Moreover, to determine whether ICAM-1 phosphorylated by c-MET is involved in the activity of SRC, the cells were treated with the c-MET ligand; HGF. Consequently, we observed that p-SRC levels increased with HGF treatment but decreased when ICAM-1 expression was silenced (Fig. 4E). Additionally, western blotting and SRC kinase assay also revealed that activity of SRC was significantly increased when ICAM-1 and c-MET were co-expressed (Fig. 4F, G). We performed co-IP to verify whether ICAM-1, as an adapter protein, affected SRC activity and the binding between c-MET and SRC. First, we found that ICAM-1 can bind to SRC and c-MET (Fig. 4H). However, when ICAM-1 expression was suppressed, a significant reduction in c-MET and SRC binding was observed (Fig. 4I, J). In addition, through immunocytochemistry experiments, ICAM-1 and c-MET were co-localized, and it was confirmed that p-SRC increased at this time (Fig. 4K). To investigate the effect of the c-MET/ICAM-1/SRC axis in CRC, we performed transwell cell migration/invasion assay and tube formation assay, and western blotting. In all experimental results, cancer cells expressing c-MET/ICAM-1/SRC together showed the highest malignancy, and p-SRC activity was also upregulated. (Fig. 4L–N). Additionally, we confirmed that c-MET was bound to the ICAM-1 extracellular domain, and the deletion construct of ICAM-1 and soluble ICAM-1 did not significantly affect SRC activity (Supplementary Fig. S4A–D). Based on these results, we demonstrated that ICAM-1 can be phosphorylated by c-MET and is an important adapter protein that regulates SRC activity in c-MET-SRC axis.

### ICAM-1 targeted antibodies inhibit the progression of cancer

**Fig. 5:**
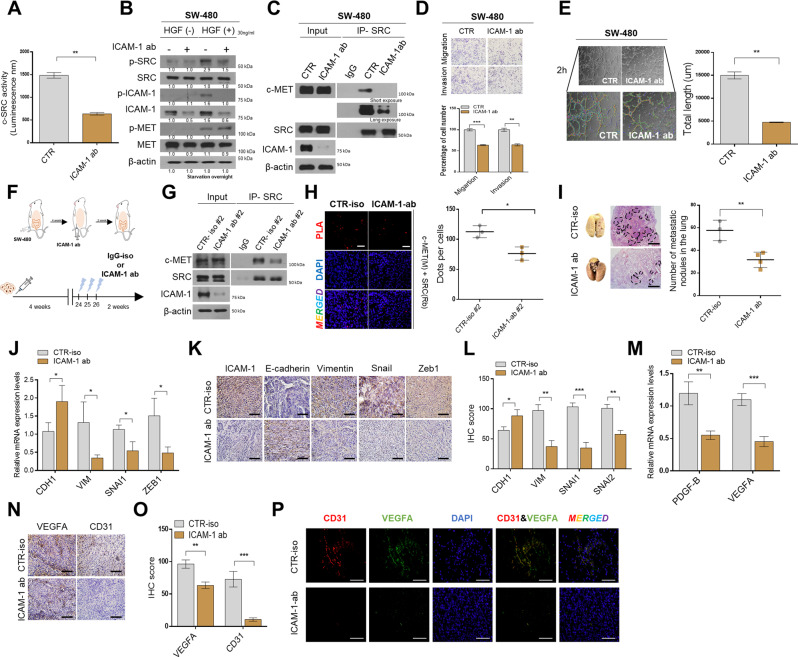
ICAM-1 targeted antibodies inhibit the progression of colon cancer. **A** Src kinase assay was detected using luminescence in SW-480 cells after 48 h treatment with ICAM-1 neutralizing antibody (2 ug/mL). **B** Western blot analysis was performed by treating SW-480 cells with ICAM-1 neutralizing antibody (2 ug/mL) for 24 h and then adding HGF (30 ng/μL) for 24 h. **C** co-IP experiment between SRC and c-MET after treatment with ICAM-1 neutralizing antibody (2 ug/mL). **D**, **E** Migration/Invasion assay and tube formation assay performed in SW-480 cells after ICAM-1 neutralizing antibody (2 ug/mL). Tube formation was assessed after 2 h using light microscopy, and the Image J program was used to analyze tube length. **F** SW-480 cells (3 × 10^6^) were injected into the cecal wall of athymic BALB/c-nude (8-week-old) mice (*n* = 3), and after 4 weeks ICAM-1 ab was intraperitoneally injected. And mice were sacrificed at 6 weeks. **G**, **H** Co-IP experiments and in situ PLA staining were performed in CTR-iso and ICAM-1-ab injected mouse tissues. **I** H&E staining images of lung tissue from mouse groups. The graph shows the number of lung metastatic lesions in each group. **J**–**L** qRT-PCR and IHC staining analysis of expression levels for EMT markers and regulators in mouse tumor tissue. **M**–**O** qRT-PCR and IHC staining aniysis of expression levels for that angiogenesis-related markers and growth in xenograft tumor tissue. **P** Representative immunofluorescence staining image for CD31 (red) and VEGFA (green) in mouse tumors. β-actin and positive control were used as a control for normalization. Data are presented as mean ± SD and analyzed by Student’s *t*-tests. *P < 0.05; **P < 0.01; ***P < 0.001.

To confirm the therapeutic effect of ICAM-1 on CRC, SW-480 cells were treated with a neutralizing antibody. As a result, it was confirmed that p-SRC activity was reduced through SRC kinase assay and western blot assay (Fig. 5A and Supplementary Fig. S5A). We also observed that p-SRC levels diminished when ICAM-1 was blocked, even after HGF treatment (Fig. 5B). In addition, neutralizing antibody treatment reduced the binding affinity between c-MET and SRC, and decreased the ability of EMT and angiogenesis, including the expression of N-CAD, VIM, Snail, Slug,VEGF-A, and PDGF-BB (Fig. 5C–E and Supplementary Fig. S5B, C). To examine the neutralizing antibody response in vivo, SW-480 cells were injected into the cecal wall of the mouse, antibodies were injected intraperitoneally after 4 weeks, and the animals were sacrificed 2 weeks later (Fig. 5F). We found that our mouse experimental results were consistent with our in vitro results. Through co-IP and PLA, we revealed that ICAM-1 blockade reduces the binding between c-MET and SRC in vivo (Fig. 5G, H). Moreover, H&E staining showed a decrease in lung metastases when ICAM-1 was blocked (Fig. 5I). In addition, as evaluated in qRT-PCR, IHC, and IF experiments on xenograft tumors, the expression of regulatory factors such as VIM, Snail, and Zeb1 was greatly reduced, and the expression of angiogenesis-promoting factors and markers was also decreased (Fig. 5J–P and Supplementary Fig. S5D–F). Therefore, we suggest that ICAM-1 neutralizing antibody serves as a novel therapeutic target because it induces the inactivation of SRC and suppresses tumor malignancy.

### Correlation between ICAM-1 expression and activity of SRC in patients with CRC

**Fig. 6:**
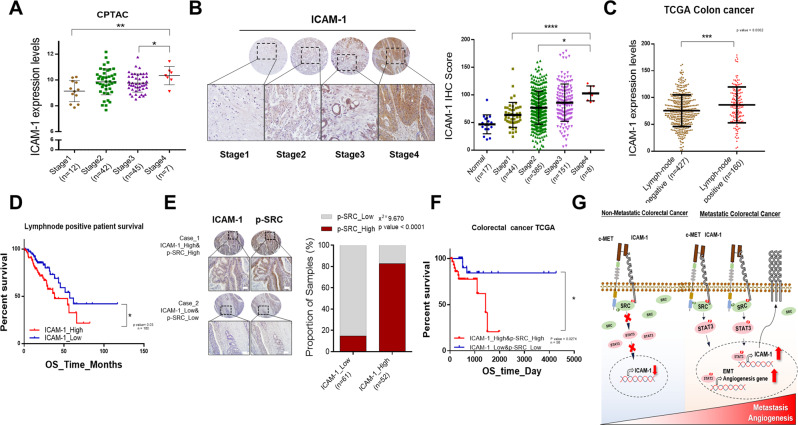
Correlation between ICAM-1 expression and SRC activity in colorectal cancer patients. **A**, **B** Analysis of ICAM-1 expression through stagewise in CPTAC database and colorectal cancer patient tissue array. High expression of ICAM-1 was positively correlated with high-stage colorectal carcinoma. Scale bar, 100 μm. **C** Lymph node metastasis incidence analysis from TCGA COAD patients. **D** Kaplan–Meier survival analysis of lymph node positive patients according to ICAM-1 expression level from TCGA COAD patients. **E** Representative IHC staining images and correlation graph between ICAM-1 and p-SRC in human colorectal cancer tissue array. Percentage of groups corresponding to statistical significance according to Student’s *t*-test and Pearson expression correlations are indicated. Scale bar = 100 μm. **F** Kaplan–Meier Survival analysis of correlation between ICAM-1 and p-SRC in the colorectal cancer TCGA database. **G** Schematic illustration of the mechanism by which ICAM-1 may act as an adapter protein of p-SRC to regulate colorectal cancer malignancy. Data are presented as mean ± SD and analyzed by Student’s *t*-tests. *P < 0.05; **P < 0.01; ***P < 0.001.

To evaluate the clinical significance of our findings, various public databases and colon tissue microarrays were analyzed according to the CRC stage. The expression level of ICAM-1 was positively correlated with disease stage and grade (Fig. 6A, B and Supplementary Fig. S6A, B). Furthermore, we found that ICAM-1 expression was higher in patients with lymph node metastases than in patients with non-lymph node metastases using the TCGA database. Moreover, in patients with lymph node metastases, Kaplan–Meier survival analysis showed that higher ICAM-1 expression had a relatively poor prognosis (Fig. 6C, D). Next, to determine if there was a correlation between ICAM-1 and p-SRC in CRC patients, we stained the tissue array. We found that ICAM-1 expression was positively correlated with p-SRC expression (Fig. 6E). Kaplan–Meier survival analysis revealed that patients with high ICAM-1 and p-SRC expression level had shorter survival rate than patients with low expression of these proteins (Fig. 6F). Collectively, our findings suggested that ICAM-1 serves as an adapter protein regulating malignancy of colon cancer. In summary, this study demonstrated a regulatory mechanism of EMT and angiogenesis by activation of SRC, which is mediated by the c-MET/ICAM-1/SRC signaling axis (Fig. 6G).

## Discussion

Cell adhesion molecules play a crucial role in cancer progression and metastasis [30]. They promote cell–cell interactions and are composed of three domains: intracellular, transmembrane, and extracellular. Based on their protein sequences and structures, cell adhesion molecules are divided into four major groups: cadherins, integrins, selectins, and immunoglobulins [31]. ICAM-1 belongs to the immunoglobulin superfamily and regulates processes such as signal transduction maintenance, cytoskeletal rearrangement, and leukocyte migration. Recent studies have shown that ICAM-1 expression is strongly correlated with poor survival and is known to promote tumor metastasis by regulating various signaling pathways in many cancer types [32]. However, only two studies have investigated function of ICAM-1 in CRC. One study showed that ICAM-1 expression levels correlated with a good prognosis [33], and another study suggested that metastasis was reduced by overexpressing ICAM-1 in their metastatic colon cell line [34]. However, these studies did not elucidate the mechanism by which ICAM-1 acts in CRC, but merely describe a simple phenomenon. Therefore, the mechanism by which ICAM-1 acts in CRC remains poorly understood and controversial.

In this study, we demonstrated that ICAM-1 has an oncogenic role in CRC. First, our results showed that higher ICAM-1 expression levels were related to poor prognosis. We also confirmed through in vitro and in vivo experiments that the level of ICAM-1 determines the migration/invasion and angiogenesis ability of the cells. Tumor aggressiveness is not regulated by the expression of only a single gene, but by the activation of various downstream signaling molecules and the expression of oncogenic transcription factors [35–37]. Likewise, ICAM-1 expression is known to promote malignancy by activating signal transductions and regulating the expression of several transcription factor [32, 38]. Therefore, we screened downstream signaling pathways to identify the mechanism by which ICAM-1 regulates malignancy in CRC. As a results, we identified that the SRC/STAT3 signaling pathway was activated by ICAM-1 and that malignancy was regulated through a rescue experiment. Additionally, we found that the expression level of ICAM-1 decreased when the signaling activities of SRC and STAT3 were suppressed. Therefore, we confirmed that STAT3 can regulate ICAM-1 expression by forming a positive feedback loop in CRC, identical to other cancer types [21–23]. Furthermore, IP analysis showed that the Tyr residue in the cytoplasmic tail of ICAM-1 plays an important role in binding ICAM-1 to SRC and in regulating its activity. However, because ICAM-1 cannot function as a kinase that directly phosphorylates proteins, we speculated that ICAM-1 would have an adapter function that could bind to SRC and indirectly regulate phosphorylation. Therefore, by screening public databases and multiple experiments, we identified that c-MET can phosphorylate ICAM-1. Moreover, we demonstrated that ICAM-1 acted as an adapter protein of the c-MET-SRC axis and affected SRC activity and binding. Finally, by treating the cells with a neutralizing antibody capable of inhibiting ICAM-1, we found decreased SRC activity, tumor metastasis, and angiogenesis. In addition, by analyzing patient data, we showed that ICAM-1 and SRC were positively correlated and affected malignancy. Consequently, we suggest that ICAM-1 could be a novel therapeutic target for CRC.

However, this study had some limitations. First, as we observed that ICAM-1 can be phosphorylated by c-MET and it contributes to the regulation of SRC activity, additional studies are needed to determine whether it can be phosphorylated by other RTKs and could modulate the activity of other kinases. Second, as ICAM-1 acted as an adapter protein to regulate the c-MET-SRC axis in CRC, further research is required to determine whether it acts as an adapter protein in other types of cancer.

In summary, we identified ICAM-1 as a metastatic marker in CRC. Furthermore, we identified the mechanism by which ICAM-1 regulates EMT and angiogenesis via the c-MET/SRC/STAT3 axis. Therefore, upregulated expression of ICAM-1 is associated with CRC malignancy. We propose that ICAM-1 could be a novel therapeutic target for the disease.

## Materials and methods

### Material and chemical reagents

The human p-CMV-ICAM-1 vector was purchased from sinobiological, pc-DNA3-SRC vector was purchased from Addgene, and p-CMV6-c-MET vector was purchased from origene. HGF (100-39H) was purchased from Peprotech. SU11274 (c-MET inhibitor), WP1066 (573097), and PP2 (529573) were obtained from Calbiochem. Antibodies specific for CDH2 (#610920) was purchased from BD Biosciences (San Jose, CA, USA). Antibodies specific for Snail(#3879), Slug (#9585), TWIST (#46702),SRC (#2108), p-SRC (#2105), MET (#3127), p-MET (#3077), and HA (#2367) were purchased from Cell Signaling Technology (Danvers, MA, USA). Antibodies specific for Vim (ab137321), CDH1 (ab1416), PDGFB (ab23914), VEGFA (ab46154), FGF2 (ab92337), STAT3 (ab226942), p-STAT3 (ab32143), ZEB1 (ab223688) and ICAM-1 (ab2213) was obtained from Abcam (Cambridge, UK). The monoclonal antibodies specific for β-Actin (SC-47778) was purchased from Santa Cruz. The monoclonal antibodies specific for FLAG (F-3165) was purchased from Sigma. The polyclonal antibodies specific for p-ICAM-1 (CSB-PA000547) was purchased from CUSABIO TECHNOLOGY.

### Cell culture and transfection

Colon cell lines (CCD-841, SW-403, H-508, HT-29, LS-174T, HCT-116, SW-480) were purchased from ATCC and incubated at 37 °C with 5% CO2 incubator. The HEK293T cells were purchased from the Korean Cell Line Bank. HEK293T cells were grown in DMEM, while colon cell lines were grown in RPMI medium. All media were supplemented with 10% fetal bovine serum, penicillin (100 U/mL), and streptomycin (100 μg/mL). All cell culture media, fetal bovine serum, penicillin/streptomycin, and trypsin were purchased from Gibco. Cells were sub-cultured by trypsin EDTA treatment. ICAM-1, SRC, and c-MET vectors or siRNAs were transfected into the cells using Lipofectamine-2000 Reagents (Invitrogen). All siRNAs were purchased from Genolution Pharmaceuticals.

### Migration and invasion assay

For migration assay, SW-480 (5 × 10^4^) and HT-29 (6 × 10^4^) cell were seeded on the upper well of transwell chamber (Coring) with 200 μL of serum-free medium, and 800 μL RPMI medium with 10% FBS was filled bottom chamber. For invasion assay, transwell chamber was pre-coated with growth factor–reduced Matrigel (BD Biosciences). Cells were cultured using the same migration assay protocol. After 2 days, migrated cells were stained using a Diff-Quick Kit (Thermo Fisher Scientific).

### Tube-formation assay

HUVEC cell was purchased from ATCC and incubated in EGM-2 medium (Lonza) with growth factor. Twenty-four-well plate bottom was pre-coated as growth factor–reduced Matrigel (BD Biosciences) in a 37 °C incubator for 30 min, and HUVEC (1 × 10^5^) cells were seeded into the coated wells. SW-480 (5 × 10^4^) and HT-29 (6 × 10^4^) cell were seeded on the upper well of transwell chamber (Coring) with 200 μL of RPMI. After incubation for 2 h, endothelial cell tube formation was captured using a microscope and analyzed with the Image J program.

### Western blotting

Cell proteins were extracted by using the lysis buffer [Tris–HCl (40 mM, pH 8.0), NaCl (120 mM), and Nonidet-P40 (0.1%)] supplemented with protease inhibitor. Proteins were separated by SDS-PAGE and transferred to a nitrocellulose membrane (Amersham). The membrane was blocked with 5% dry-milk in PBST and incubated with primary antibody in 2.5% BSA (Bovogen) buffer, and then the membrane was incubated with an HRP-conjugated secondary antibody. Finally, chemiluminescence (Amersham) was used to detect the protein.

### Co-immunoprecipitation

Lysates were prepared with lysis buffer as described in western blotting. After quantifying the protein levels, the lysates were incubated overnight with the primary antibody. The next day, protein A-agarose beads (Santa Cruz) were added to the sample tube and incubated for 2 h at 4 °C in an orbital shaker, and the protein A-agarose beads were washed three times with cold PBS and followed by Western blotting analysis.

### Antibody arrays

The Human Phospho-Kinase Array Kit was purchased from R&D Systems (ARY003B). Cell lysates were prepared such as Western blotting protocol and then manufacturer’s instructions were followed.

### Immunocytochemistry and proximity ligation assay

Cultured cells on a cover slip were fixed with 4% paraformaldehyde, and permeabilized with 0.1% Triton in PBS. Cells were blocked with 5% normal goat serum in PBS and were incubated overnight with the primary antibody. Immunocytochemistry was incubated with 488- or 594-conjugated secondary antibody (Molecular Probes) for 2 h, and DAPI (Sigma-Aldrich) was used to counterstain the nuclei. In situ PLA was performed with the manufacturer’s protocol using the Duolink Detection Kit. Data were analyzed using a confocal microscopy and quantified using the Image J program.

### RNA extraction and qPCR

RNA was isolated using the Trizol (Invitrogen), and RNA quality was measured using a NanoDrop Spectrophotometer (ND1000, NanoDrop Technologies). qRT-PCR was performed using the KAPA SYBR FAST qPCR Kit (KAPA Biosystems) and the manufacturer’s instructions were followed. The reaction was performed on Rotor-Gene Q (Qiagen), and the results were expressed as fold-changes calculated using ΔΔCt method. β-actin was used as an internal normalization control. All primers were purchased from DNA Macrogen. All primers used in this study are listed in Supplementary Table.

### SRC activity assay and HA-tagged protein purification kit

The lysates quantified were incubated overnight with the c- SRC antibody. After overnight, protein A-agarose beads (Santa Cruz) were placed in a sample tube and incubated for 2 h at 4 °C in an orbital shaker. 0.1–0.2 M glycine (pH2.0–3.0) buffer was added to the sample tube to attenuate the interaction between the antibody and the bead. For neutralization, 1 M Tris buffer (pH8.0) was added to the sample tube in an equal volume. The SRC activity was measured by quantifying the amount of ADP produced during kinase using the SRC kinase enzyme system (Promega, V2921) and ADP-Glo kinase assay (Promega, V9101). The HA-tagged protein purification kit was purchased from MBL INTERNATIONAL (3320). Cell lysates were prepared such as then manufacturer’s instructions were followed.

### Point mutation and cloning

The point mutant structure of the ICAM-1 tyrosine residue was generated using pfu-X DNA Polymerase (Solgent, SPX16-R250) and performed according to the manufacturer’s protocol. The mutant form of DNA was transformed with DH5α and the plasmid DNA sequence was analyzed using the Macrogen company’s DNA sequencing service. For the generation of deletion mutant of ICAM-1 domain, PCR was performed. In the first PCR step, an insert DNA was made, and was denatured at room temperature, and then the deleted DNA was amplified through the final PCR step. The insert and backbone DNAs were digested using the restriction enzyme, followed by ligation and transformation step. Primer sequence used for point mutations and deletion mutations are listed in Supplementary Table.

### Immunohistochemistry

Mice tissues (lung, liver, and Xenografts) were fixed in 9% formalin for the preparation of paraffin blocks. Paraffin-embedded tissue sections were deparaffinized in xylene, and then rehydrated in 95%, 90%, 80%, 70% ethanol. Antigen retrieval was obtained with 1 mM EDTA, and next permeabilization step was used 0.5% Triton X-100. Sections were stained with hematoxylin & eosin (H&E) or blocked with 5% normal goat serum and 3% bovine serum albumin. Next, mouse tissues incubated overnight with primary antibodies. After washing with PBS, biotinylated goat anti-rabbit IgG or anti-mouse IgG antibody was applied to the sections for 1 h at room temperature. The ABC reagent (Vector Laboratories Inc., Burlingame, CA, USA) was applied to the sections for 1 h. Color development was performed using 3,3′-diaminobenzidine (Vector Laboratories) staining. Finally step, the tissue samples were dehydrated in 70%, 80%, 90%, 95% ethanol and xylene. The mounting solution was used a Synthetic mountant (Thermo).

### In vivo xenografts and metastasis models

The SW-480 cell line was engineered for ICAM-1 knockdown using a lentiviral system and sorted 2–3 times using puromycin (Invivogen). The males of athymic BALB/c-nude (8-weeks old) mice were obtained from Orient Bio. For these xenograft experiments, 40 μL of colon cell line (3 × 10^6^) was injected into the cecal wall of BALB/c-nude mice and the mice were sacrificed 6 weeks after injection. ICAM-1 specific monoclonal antibody (GTX20020) was obtained from Genetex. The ICAM-1 ab (1.25 mg/kg) was injected intraperitoneally. For the metastasis model system, males of NSG (6 weeks old) mice were obtained from Jackson Lab. Also, 100 μL of colon cell line (1 × 10^6^) was injected into tail vein of NSG mice and the mice were sacrificed 6 weeks after the injection.

### Human tissue microarray

Colon tissue microarray samples were obtained from US Biomax (CO6161). Healthy specimens were also included in each of the array blocks. Samples were reviewed by a pathologist to confirm the diagnosis of colon carcinoma, grade, and stage. Staining density was analyzed with the Image J program.

### Chromatin immunoprecipitation assays

ChIP assays were performed using an EZ-ChIP Kit (EMD Millipore) according to the manufacturer’s instructions. Immunoprecipitation was performed using an anti-p-STAT3 antibody or a rabbit isotype control IgG (Upstate Biotechnology).

### GEO series and the cancer genome Atlas database, gene set enrichment analysis,

The microarray datasets (GSE41258, GSE4183, GSE44076, GSE9348, GSE15960, GSE17538) were downloaded from GEO to analyze the expression levels of various gene in cancer. The survival, phosphorylation data from the TCGA database were downloaded. The stage data of colorectal cancer patient were obtained from the Clinical Proteomic Tumor Analysis Consortium. Gene set enrichment analysis (GSEA) was performed by reanalyzing GSE4183, GSE44076.

### Statistical analysis

All data are presented as the means ± standard deviations (SDs). Each experiment was repeated at least three times. The statistical significance of all experimental data was determined with two-tailed Student’s *t*-test. In all instances, *P* < 0.05 was considered significant, variances were confirmed to be similar between groups that were being statistically compared. Data analyses were done with Prism software. Significance was defined as *P < 0.05, **P < 0.01, and ***P < 0.001 compared to controls. Non-significance was denoted as n.s.

## Supplementary information

Supplementary figure legend

raw data_western

raw data_staining

method check list

## Data Availability

Other datasets used in this study are publicly available in The Cancer Genome Atlas Program and GEO database (GSE41258, GSE4183, GSE44076, GSE9348, GSE15960, GSE17538).
