# Correction: ICAM-1 promotes cancer progression by regulating SRC activity as an adapter protein in colorectal cancer

**DOI:** 10.1038/s41419-022-05425-0

**Published:** 2022-11-18

**Authors:** Eun-Ji Lim, Jae-Hyeok Kang, Yeon-Ju Kim, Seungmo Kim, Su-Jae Lee

**Affiliations:** Department of Life Science, Research Institute for Natural Sciences, Hanyang University, Seoul, 04763 Korea

**Keywords:** Metastasis, Oncogenes

Correction to: *Cell Death and Disease* 10.1038/s41419-022-04862-1, published online 29 April 2022

The original version of this article unfortunately contained a mistake. The authors found that the number was entered incorrectly in the Acknowledgements part of the written article. The number “2020R1A4A101839812” should be modified to “2020R1A4A1018398”. The original article has been corrected.

