# Supplementary material for: ICAM-1 promotes cancer progression by regulating SRC activity as an adapter protein in colorectal cancer
Source: Cell Death Dis. 2022 Apr 29;13(4):417. doi: 10.1038/s41419-022-04862-1 (PMC9054780; doi:10.1038/s41419-022-04862-1)
Supplement: Supplementary file 1 — Supplementary figure legend [file 41419_2022_4862_MOESM1_ESM.docx]

**Supplementary figure legend**

**
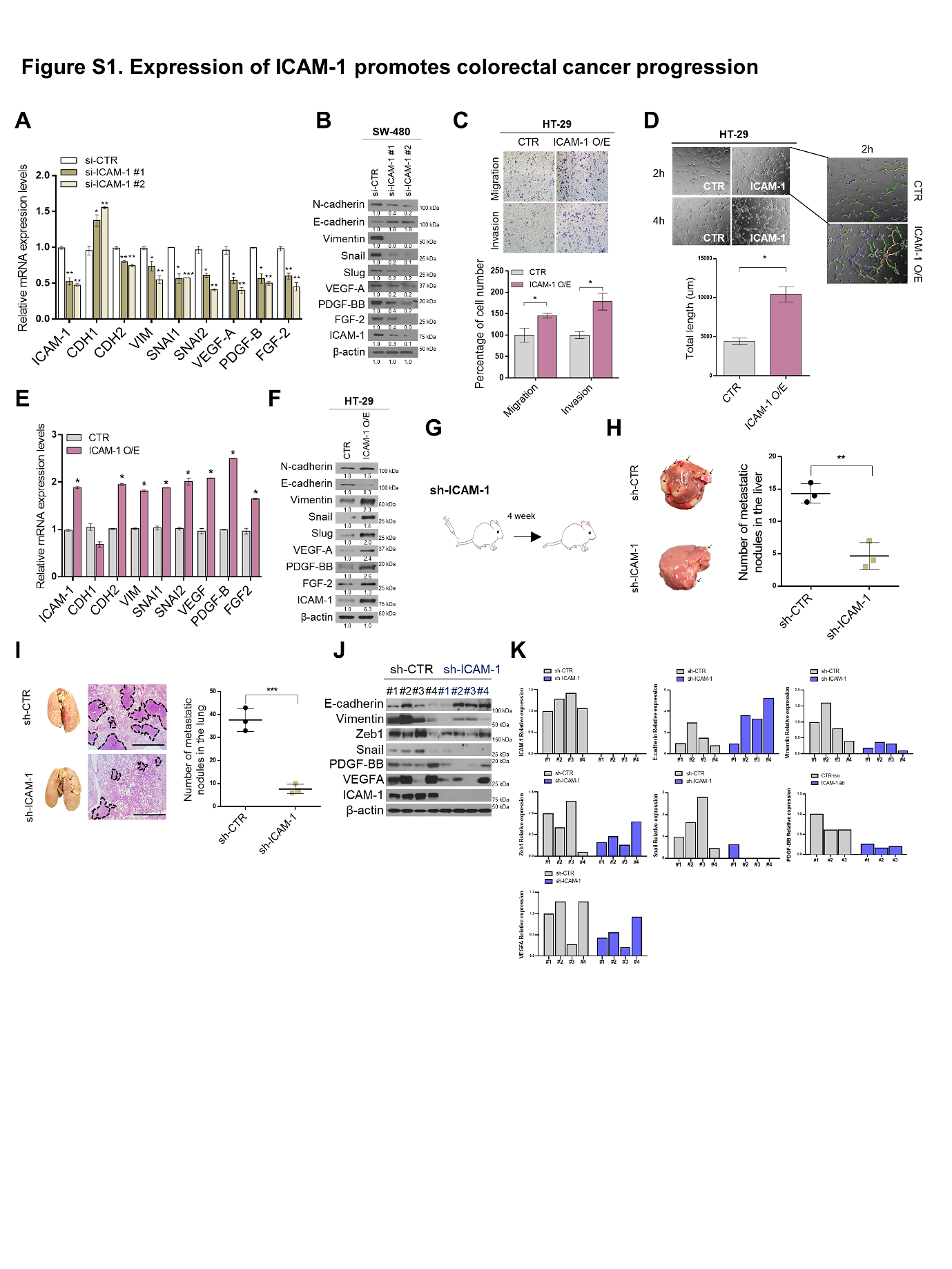
**

**Figure S1.** Expression of ICAM-1 promotes colorectal cancer progression. (**A, B**) qRT-PCR and Western blotting analysis of the expression of EMT and angiogenesis-related genes after ICAM-1 knockdown in SW-480 cells. (**C**) Migration/Invasion assay. (**D**) The tube formation assay of HUVEC cells was performed by incubation with HT-29 cells overexpressing ICAM-1. (**E-F**) qRT-PCR and Western blotting analysis of EMT and angiogenesis-related genes after ICAM-1 overexpression in HT-29 cells. (**G**) SW-480 cells transfected with sh -CTR and sh-ICAM-1 (1X 106) were injected into the tail vein of and NOD/SCID gamma (NSG, 5–6 weeks old) mice (n=4). (**H, I**) Representative H&E staining images of lung and liver metastases. And the graph shows the number of metastatic lesions in each mouse. (**J,K**) Western blotting analysis of the expression of EMT markers and regulator in xenograft models. Data are presented as mean ± SD and analyzed by Student’s t-tests. *P < 0.05; **P < 0.01; ***P < 0.001.

**
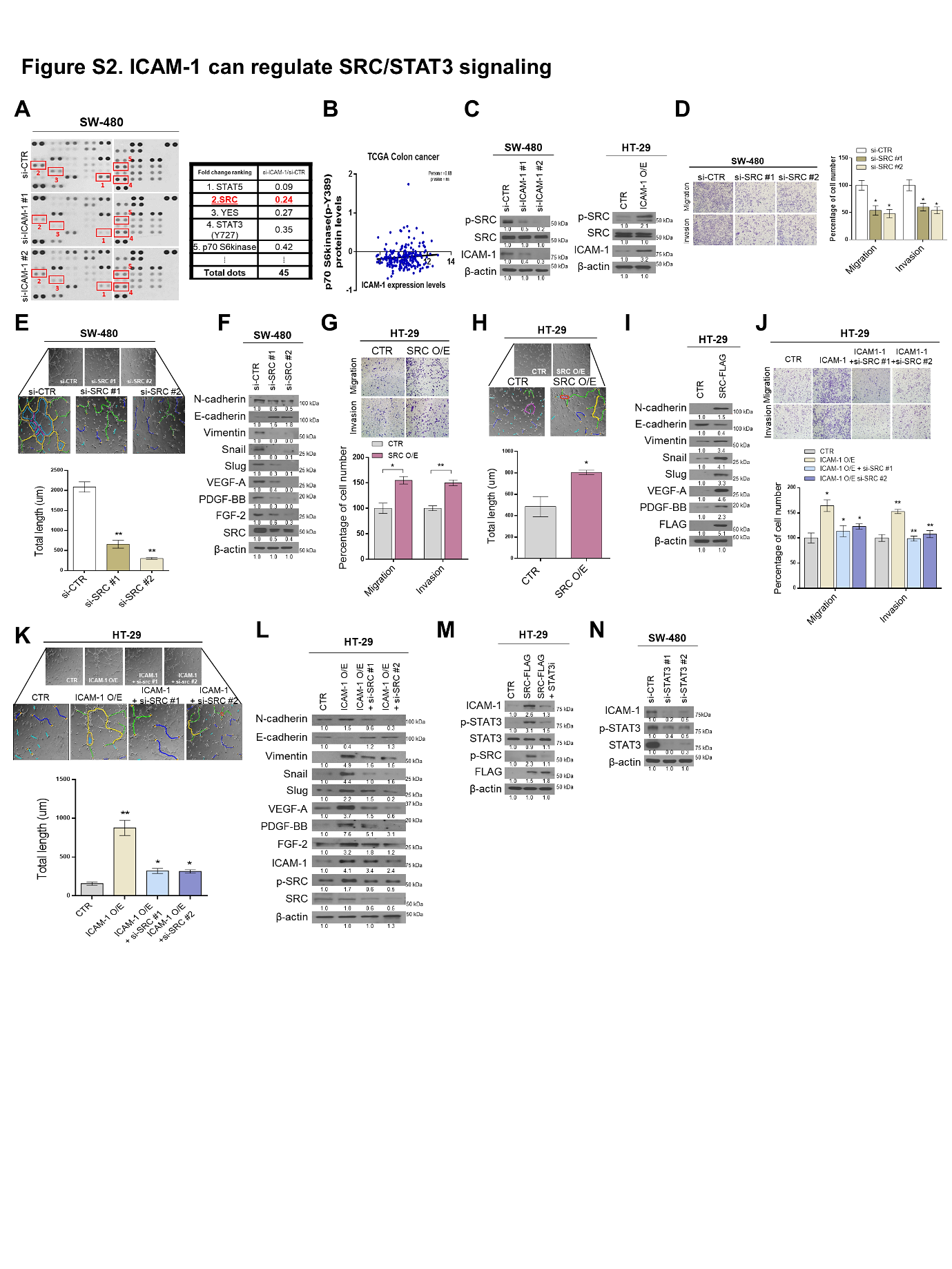
Figure S2.** ICAM-1 can regulate SRC/STAT3 signaling. (**A**) The whole blot image of kinase assay. (**B**) Pearson correlation analysis between ICAM-1 and p70 S6kinase in TCGA colon cancer patient cohort. (**C**) Western blot analysis of p-SRC according to ICAM-1 expression level in SW-480 and HT-29 cells. (**D-F**) All phenomena were found to decrease when the expression of SRC was silenced through transwell chamber analysis, tube formation analysis, and Western blotting. (**G-I**) In contrast, all experimental results were increased when SRC was overexpressed. (**J**) Migration and invasion assays were performed using HT-29 cells with overexpressed ICAM-1 and knockdown SRC. (**K**) Tube formation analysis of rescue experiments in HT-29 cells. Tube formation was assessed after 2 h using light microscopy, and the Image J program was used to analyze tube length. (**L**) Western blotting of EMT and angiogenesis-related genes in HT-29 cells by rescue experiments. (**M**) Western blotting in HT-29 cells after treatment with with overexpressed SRC and STAT3 inhibitors. (**N**) western blotting for ICAM-1 expression when STAT3 is silenced in SW-480 cells.

Data are presented as mean ± SD and analyzed by Student’s t-tests. *P < 0.05; **P < 0.01; ***P < 0.001.

.

**
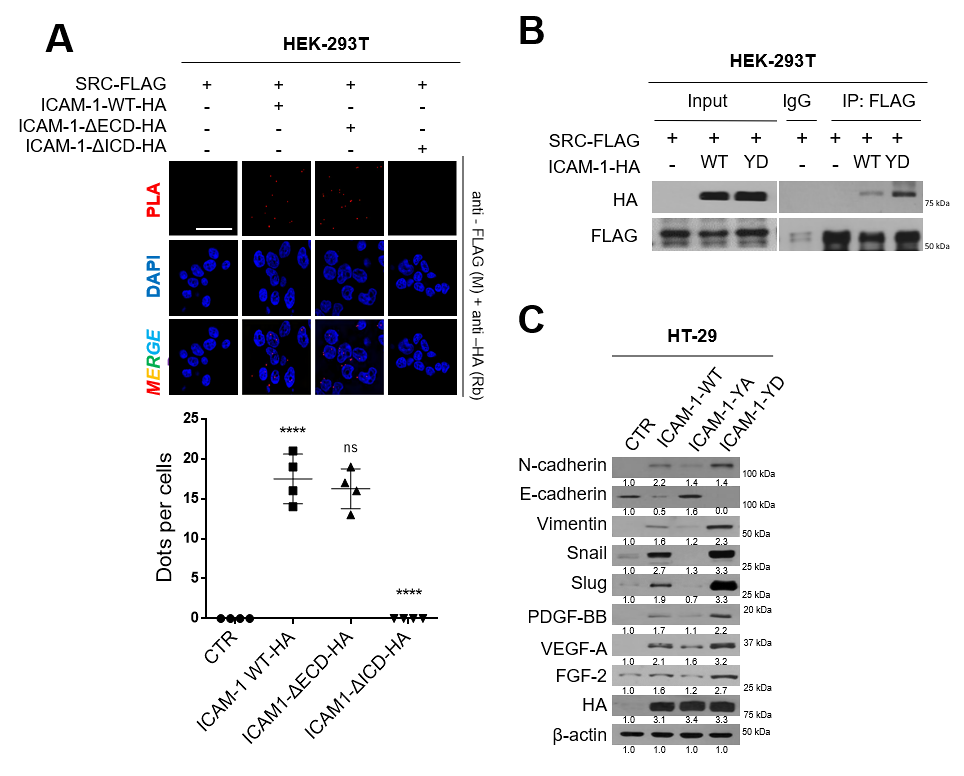
**

**Figure S3.** The Tyr 512 residue of ICAM-1 regulates EMT and angiogenesis through SRC signaling. (**A**) Representative images of in situ PLA staining the interaction between ICAM-1 deletion construct and SRC. The graph shows the number of dots per cell counted using ImageJ software. Scale bar = 100 μm. (**B**) Co-IP assay showing protein interactions of ICAM-1 point mutation structure and SRC in HEK-293T cells. Tyrosine-to-aspartic acid (Y512D) substitution increases binding to SRC compared to wild-type. (**C**) Western blot analysis of the expression of EMT and angiogenesis-related proteins in HT-29 cells transfected with various ICAM-1 point mutation constructs. Data are presented as mean ± SD and analyzed by Student’s t-tests. *P < 0.05; **P < 0.01; ***P < 0.001.

**
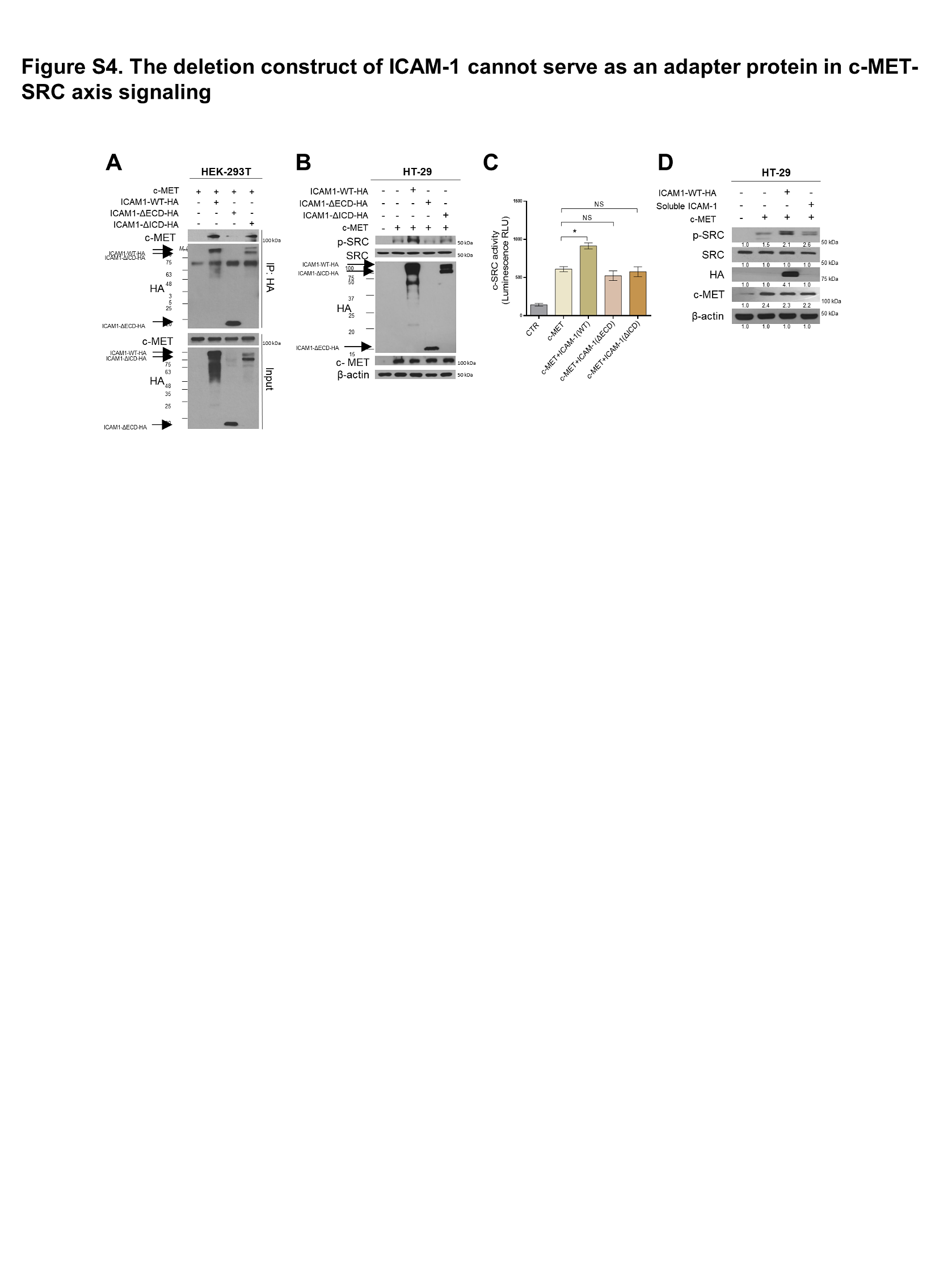
**

**Figure S4.** The deletion construct of ICAM-1 cannot serve as an adapter protein in c-MET-SRC axis signaling. (**A**) Co-IP assay is showed that c-MET binds to the extracellular domain of ICAM-1. HEK-293T cells were co-transfected with expression plasmids encoding c-MET and deletion construct of HA-ICAM-1. (**B.C**) Western blot analysis and SRC activity analysis were performed in HT-29 cells transfected with the ICAM-1 deletion construct. The deletion construct of ICAM-1 cannot regulate the activity of SRC. (**D**) Western blot analysis of the effect on soluble-ICAM-1 compared to wild-type ICAM-1. Data are presented as mean ± SD and analyzed by Student’s t-tests. *P < 0.05; **P < 0.01; ***P < 0.001.

**
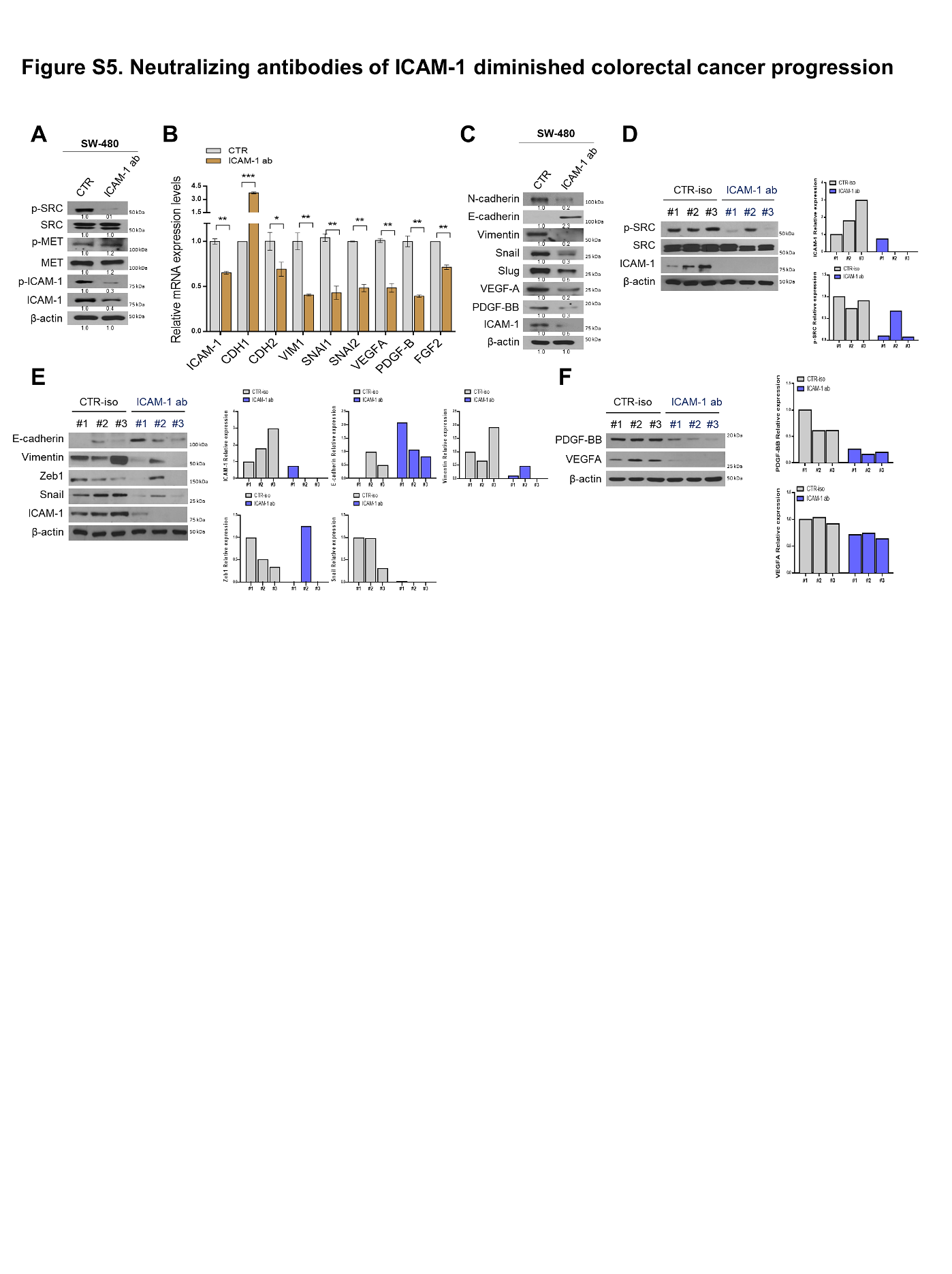
Figure S5.** Neutralizing antibodies of ICAM-1 diminished colorectal cancer progression. (**A**) Western blot analysis of effects on ICAM-1 neutralizing antibodies. (**B-C**) qRT-PCR and Western blotting analysis of EMT and angiogenesis-related genes after ICAM-1 neutralization in SW-480 cells. (**D-F**) Western blot analysis of expression levels of p-SRC, EMT and angiogenesis-related proteins in xenograft tumor tissues. β-actin and positive control were used as a control for normalization. Data are presented as mean ± SD and analyzed by Student’s t-tests. *P < 0.05; **P < 0.01; ***P < 0.001.

**
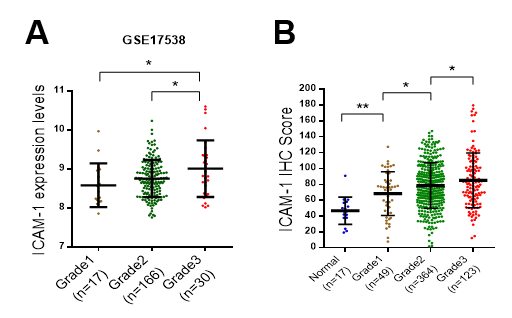
**

**Figure S6.** The expression level of ICAM-1 is positively correlated with the grade of colorectal cancer patient. (**A, B**) Analysis of ICAM-1 expression by grade in GSE 17538 datasets and colorectal cancer patient tissue array. High expression of ICAM-1 was correlated with colorectal cancer grade. Data are presented as mean ± SD and analyzed by Student’s t-tests. *P < 0.05; **P < 0.01; ***P < 0.001.
