# Supplementary material for: ICAM-1 promotes cancer progression by regulating SRC activity as an adapter protein in colorectal cancer
Source: Cell Death Dis. 2022 Apr 29;13(4):417. doi: 10.1038/s41419-022-04862-1 (PMC9054780; doi:10.1038/s41419-022-04862-1)
Supplement: Supplementary file 2 — raw data_western [file 41419_2022_4862_MOESM2_ESM.pdf]

Fig. S1

S1B

SW480: si-CTR / si-ICAM #1 / si-ICAM #2

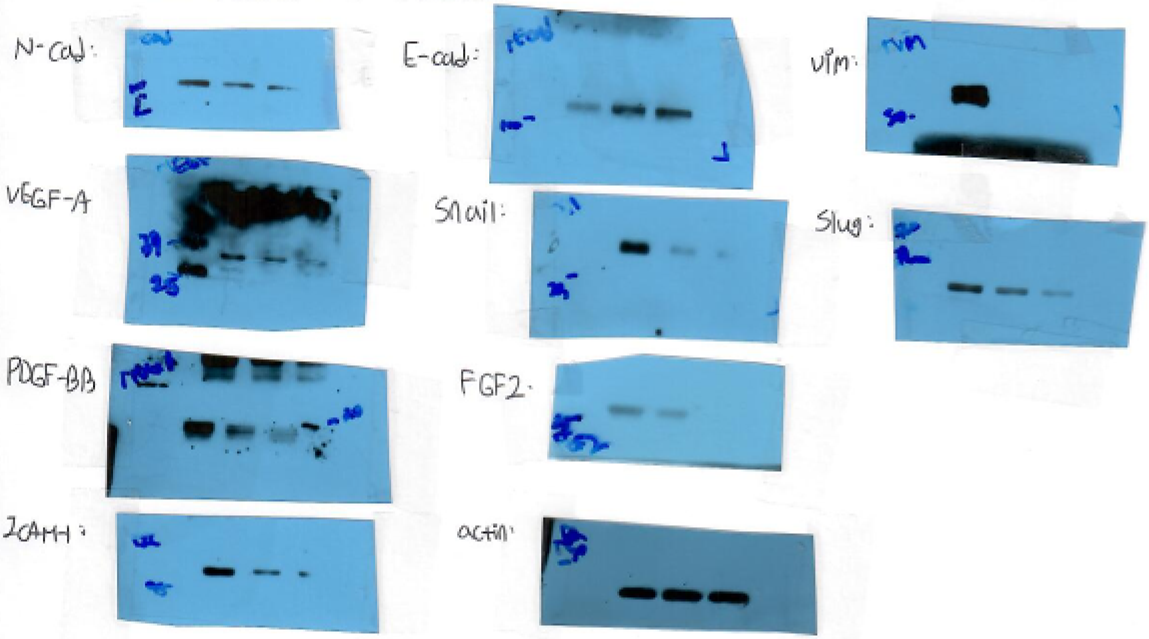

S1F

HT29: CTR / ICAM- O/E

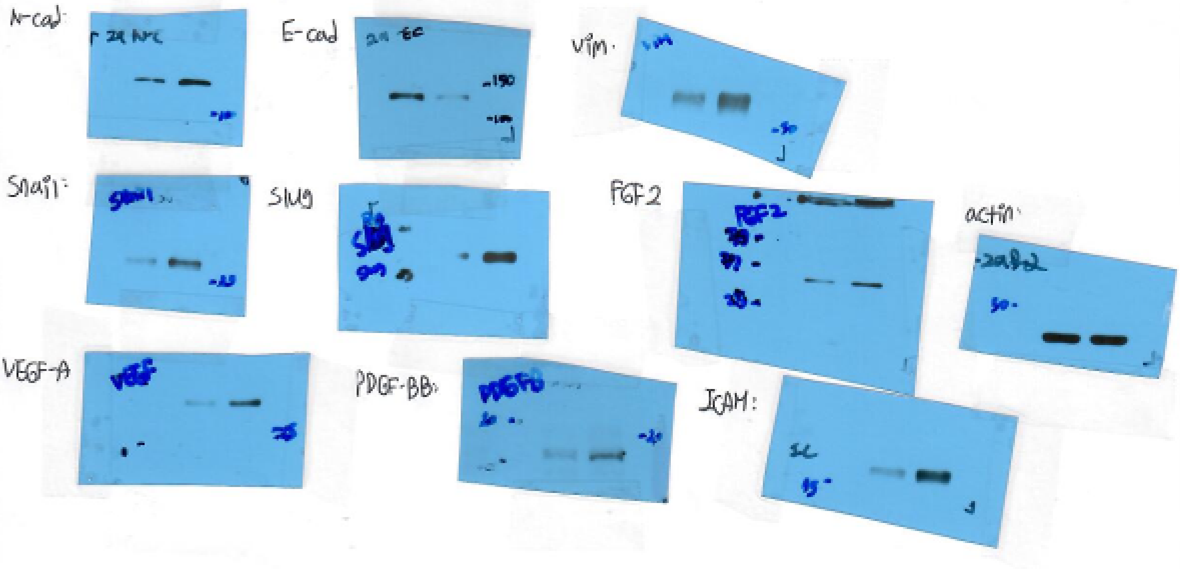

Fig. s1

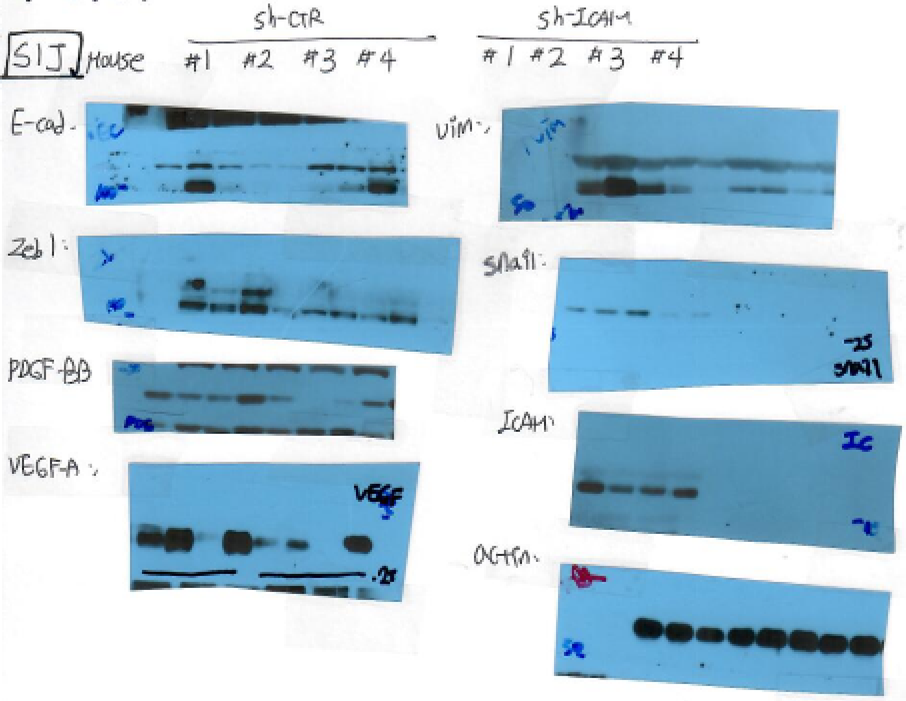
S1J
Mouse
sh-CTR
#1 #2 #3 #4
sh-ICAM
#1 #2 #3 #4
E-cad.
Zeb1:
PDGF-BB
VEGF-A:
Vim:
Snail:
ICAM:
Actin:

Fig. 2

2J: HT29: CTR | IC 0/E | IC -16 +SRCi

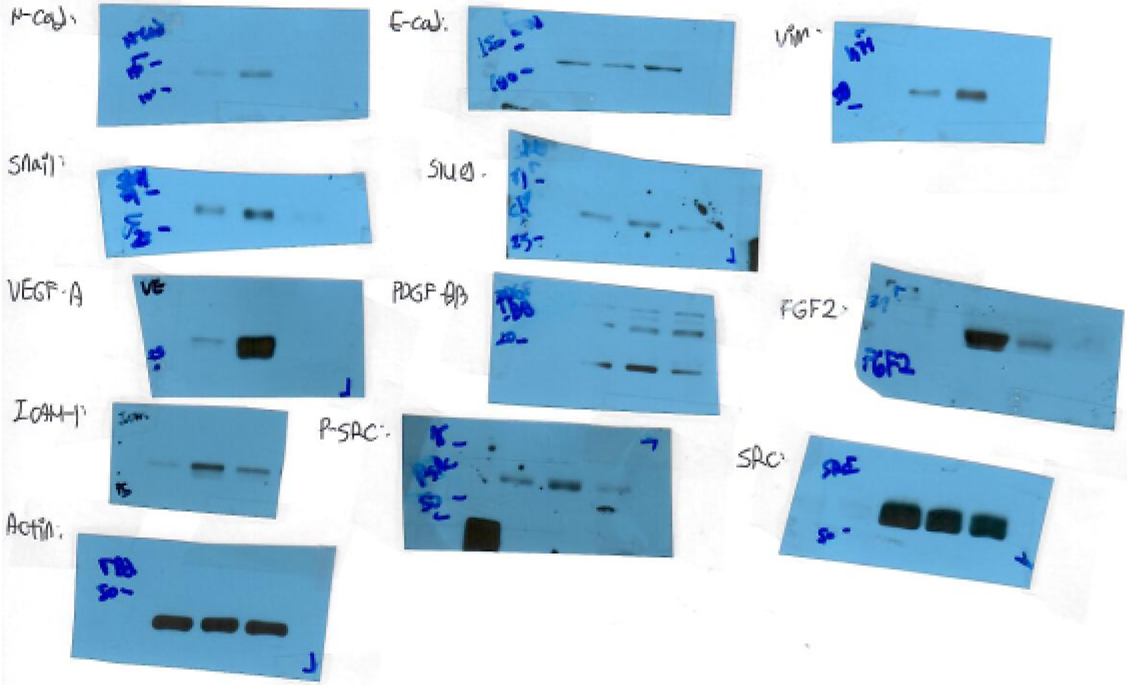

2K: HT29: CTR | IC0/E | IC0/6 + SRCi

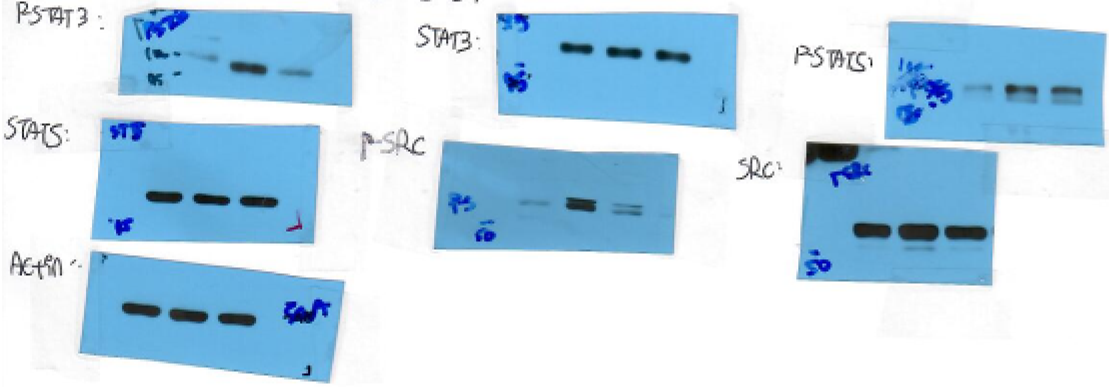

Fig.2

2N. HT29- CTR / IC o/E / IC o/E +STAT3i

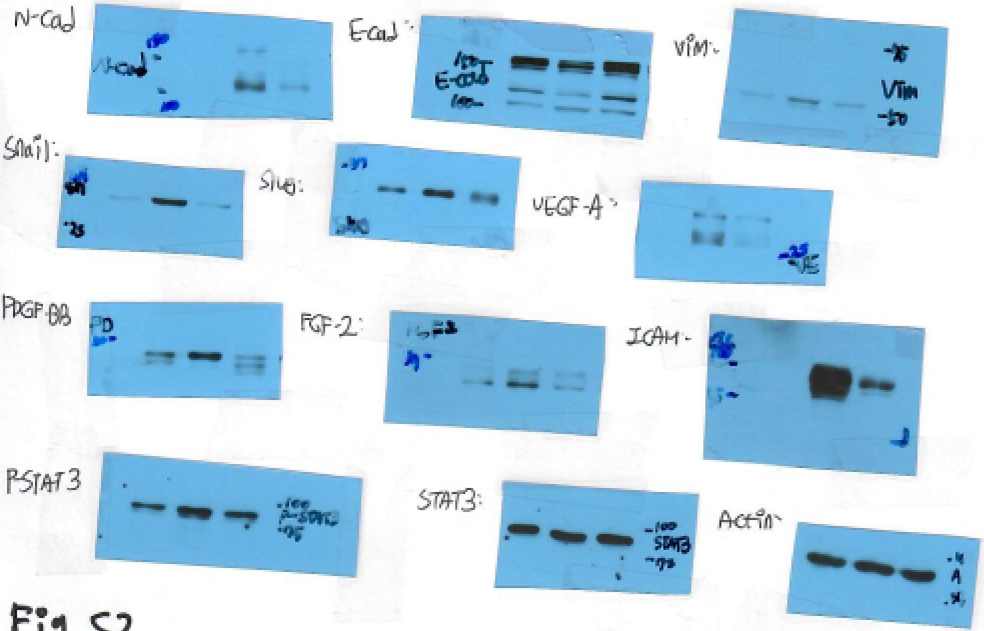

Fig. S2

2A, S2A : SW480 : CTR / si-ICAM #1 / si-ICAM #2

Magnified dot blot

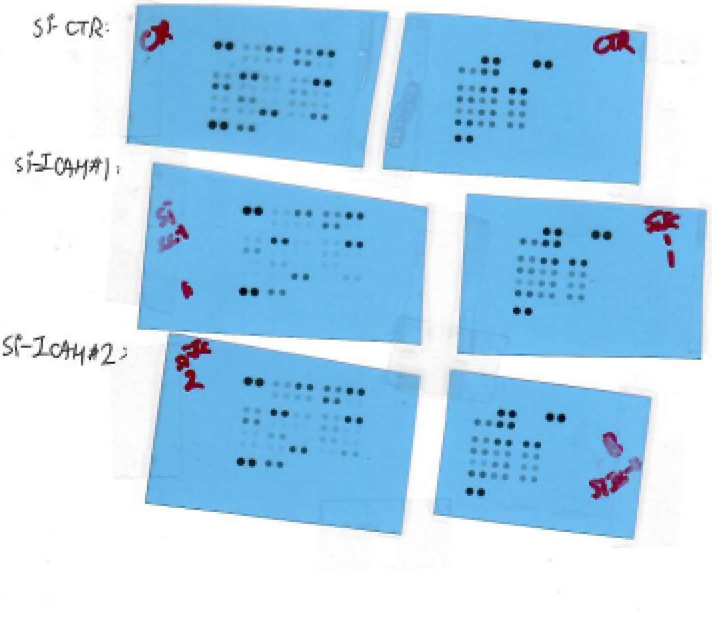

Fig. S2

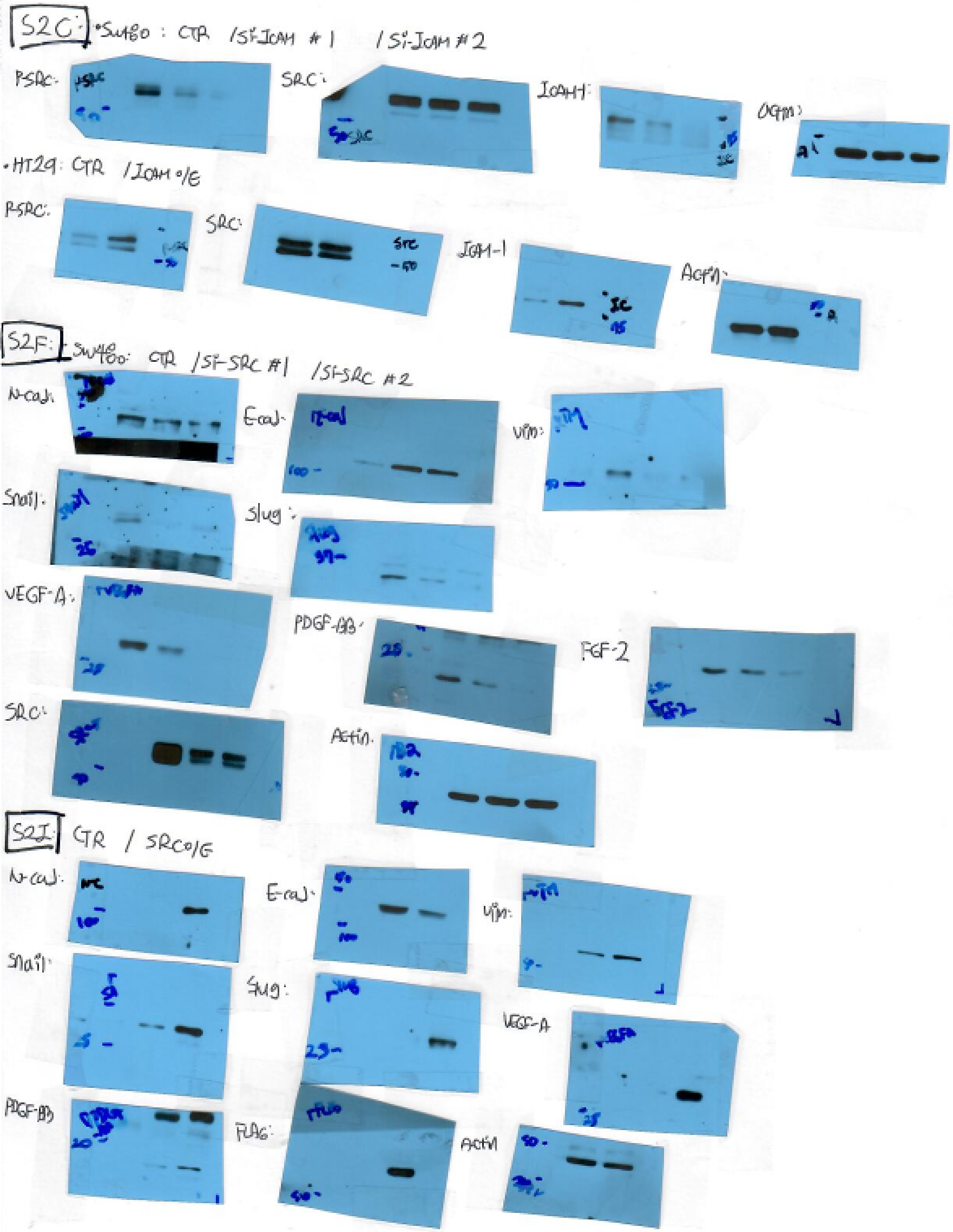
S2C:
SW480 : CTR / Si-JCAM #1 / Si-JCAM #2
PSRC:
SRC:
JCAM1:
Actin:
HT29: CTR / JCAM o/E
PSRC:
SRC:
JCAM-1
Actin:
S2F:
SW480: CTR / Si-SRC #1 / Si-SRC #2
N-cad:
E-cad:
Vim:
Snail:
Slug:
VEGF-A:
PDGF-BB:
FGF-2
SRC:
Actin:
S2I:
CTR / SRC o/E
N-cad:
E-cad:
Vim:
Snail:
Slug:
VEGF-A
PDGF-BB
FLAG:
Actin

Fig. S2

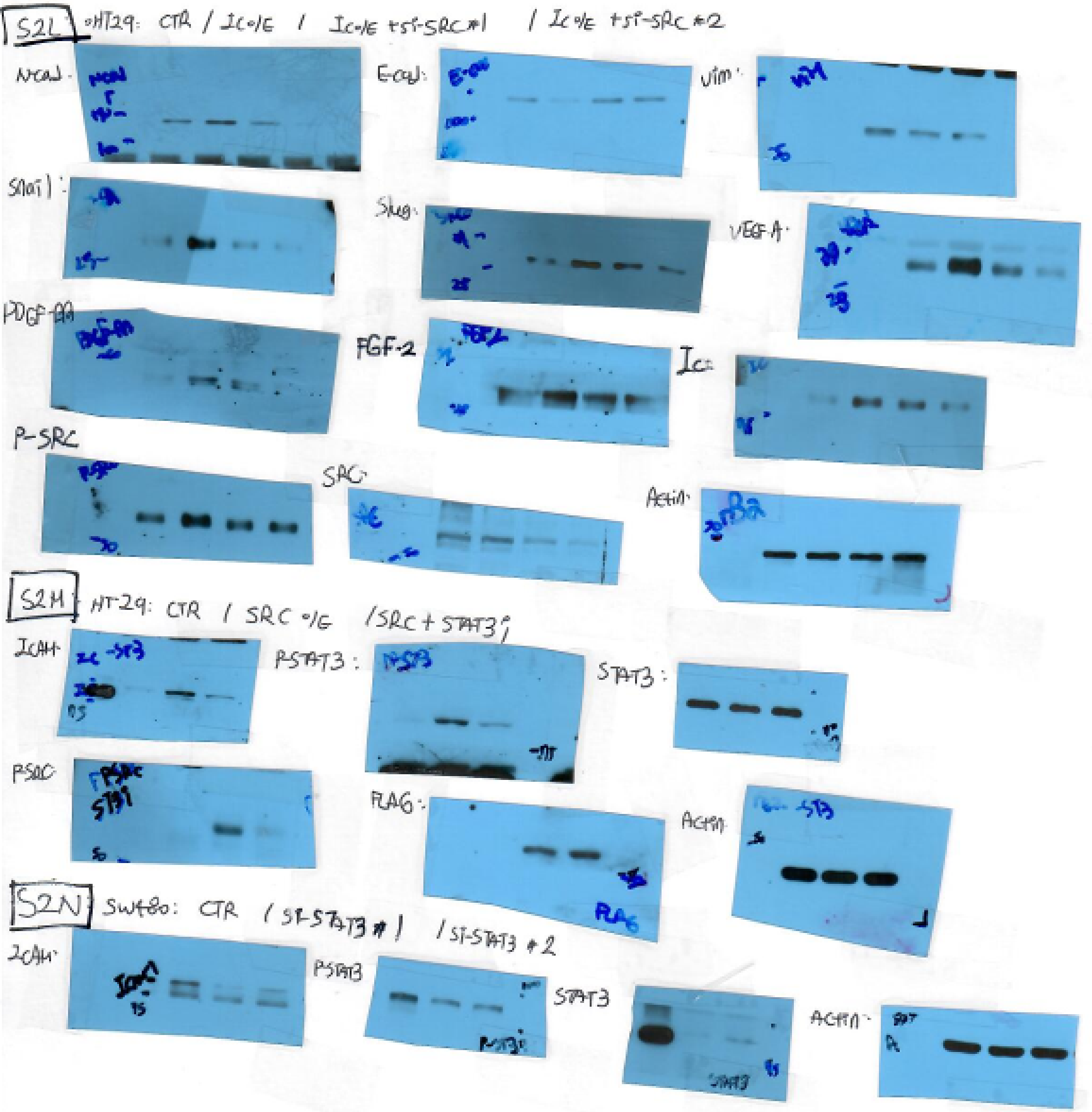
S2L
HT29: CTR / IC o/E / IC o/E + si-SRC #1 / IC o/E + si-SRC #2
Ncad
E-cad
Vim
Snail
Slug
VEGF-A
PDGF-AA
FGF-2
IC
P-SRC
SRC
Actin
S2M
HT-29: CTR / SRC o/E / SRC + STAT3 i
ICAM
P-STAT3
STAT3
PSRC
FLAG
Actin
S2N
SW480: CTR / si-STAT3 #1 / si-STAT3 #2
ICAM
PSTAT3
STAT3
ACTIN

Fig 3

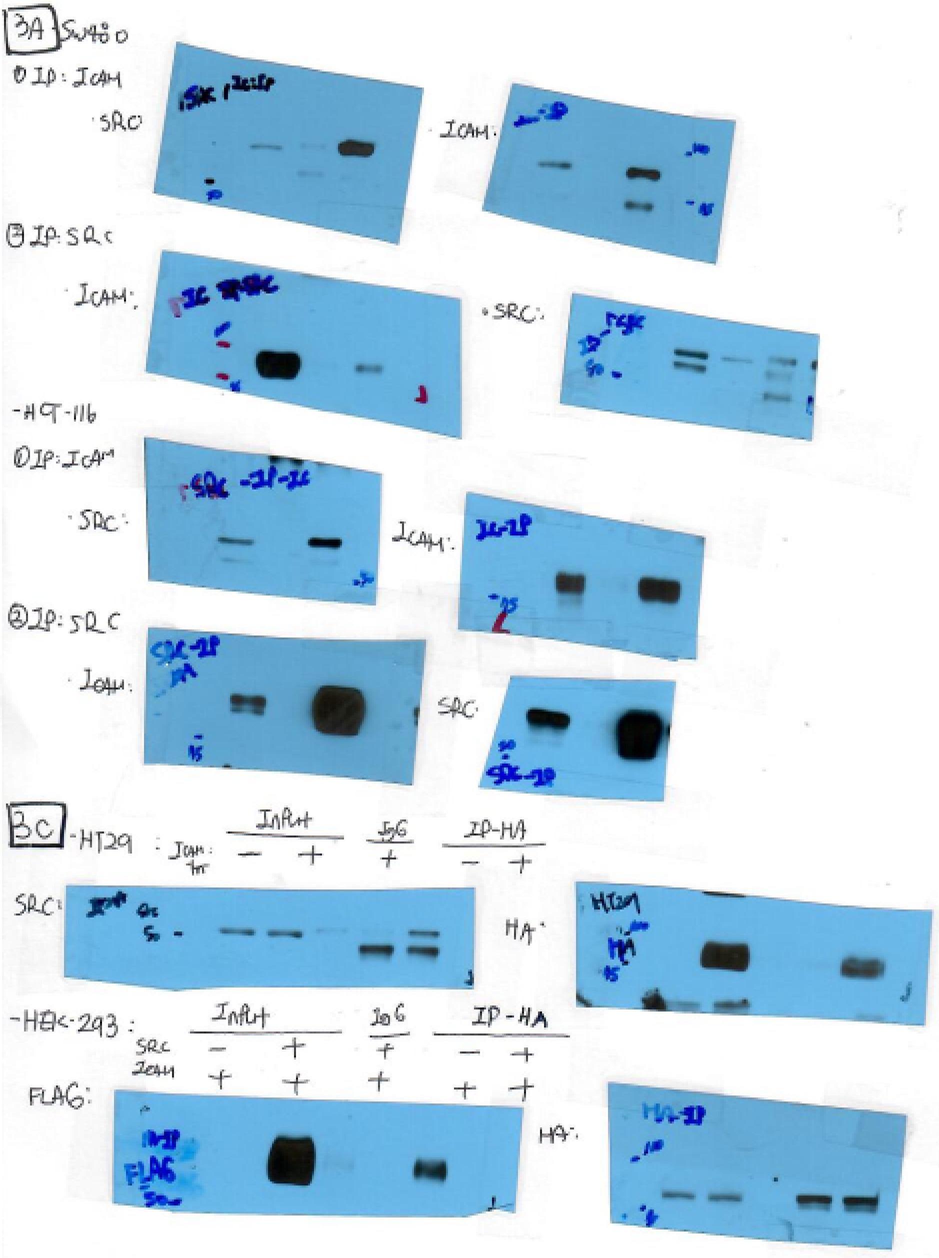
3A SW480
① IP: ICAM
SRC
ICAM
② IP: SRC
ICAM
SRC
- HCT-116
① IP: ICAM
SRC
ICAM
② IP: SRC
ICAM
SRC
3C - HT29
Input IgG IP-HA
- + + - +
SRC
HA
- HEK-293
Input IgG IP-HA
SRC - + + - +
ICAM + + + + +
FLAG
HA

Fig. 3

3D HEK293T SRC: + + + + IP: FLAG

ICAM: — WT ΔECD ΔICD

IP: HA -

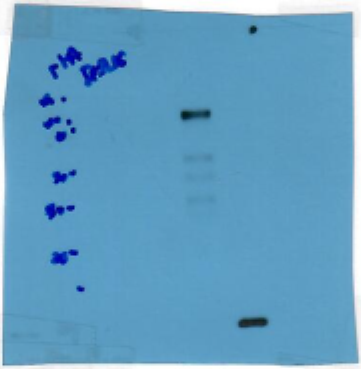

FLAG -

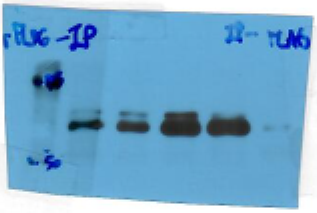

Input: HA -

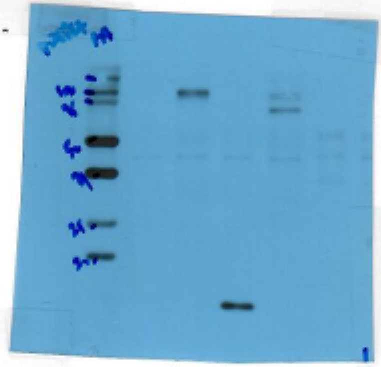

FLAG:

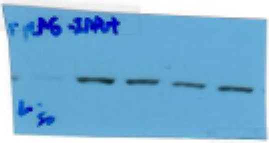

Actin -

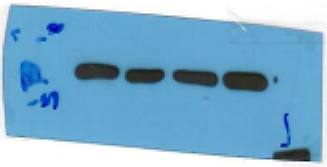

3F: HT29: ICAM: Input: — WT TA | IgG: — | IP: HA: — WT TA

SRC:

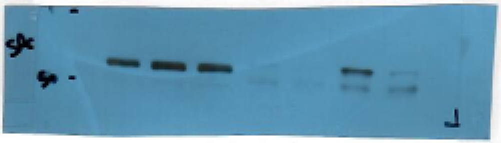

HA:

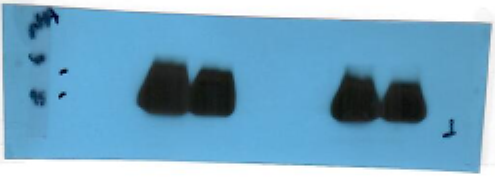

HT29: ICAM: Input: — WT YD | IgG | IP: SRC: — WT YD

Input HA:

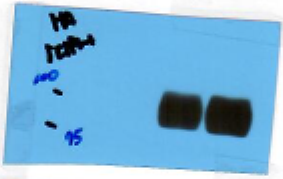

IP HA:

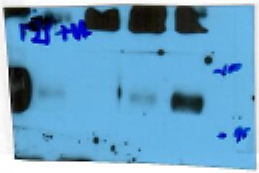

SRC

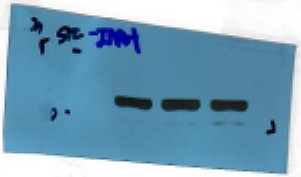

SRC:

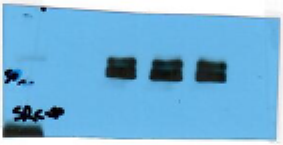

Fig. 3

3I HT29 - CTR / WT / YA / YD

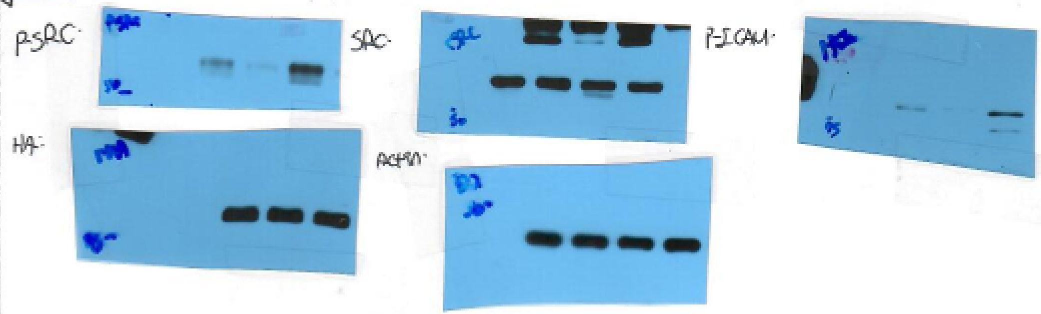

Fig. S3

S3B : HEK 293T

	INPUT			IgG	IP-FLAG		
SRC:	+	+	+	+	+	+	+
IC:	—	WT	YD	—	—	WT	YD

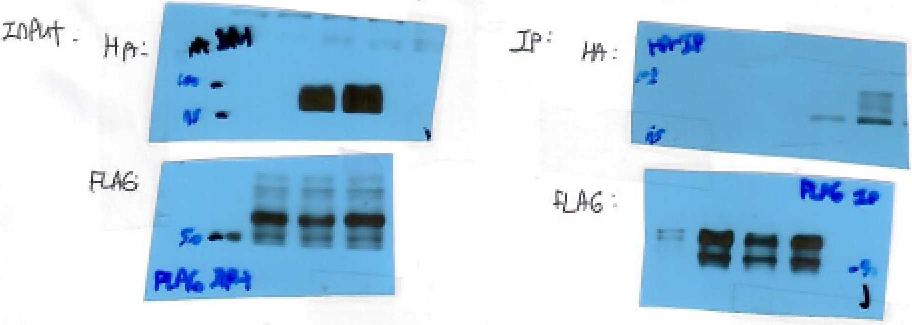

S3C HT29: CTR WT TA TD

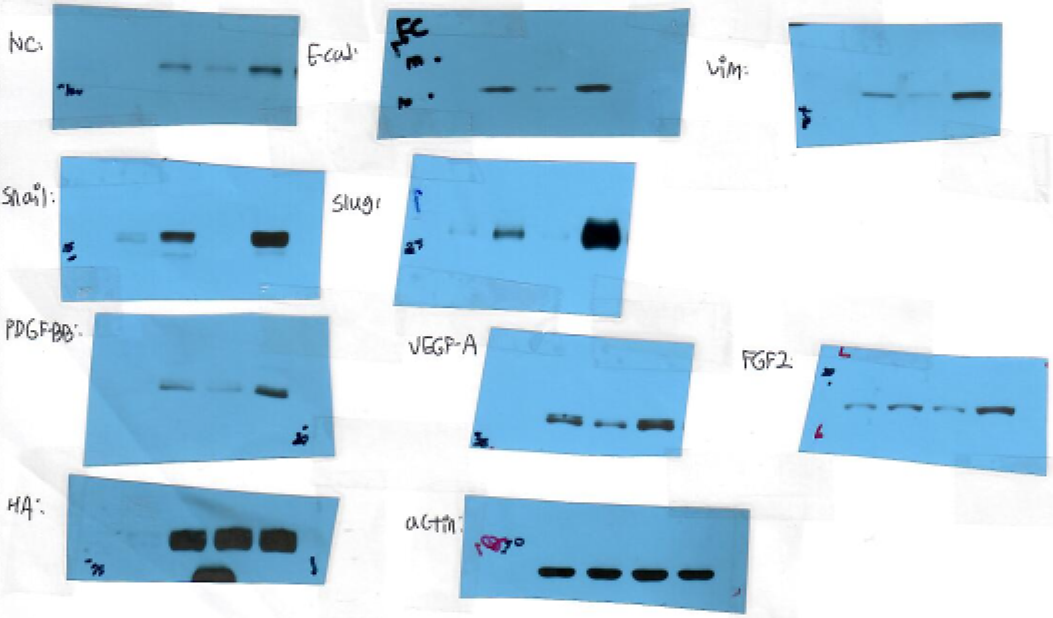

Fig. 4

4B: SW480: 0 / 0.5h / 1h / 3h / 6h / 9h / 12h - cMet i

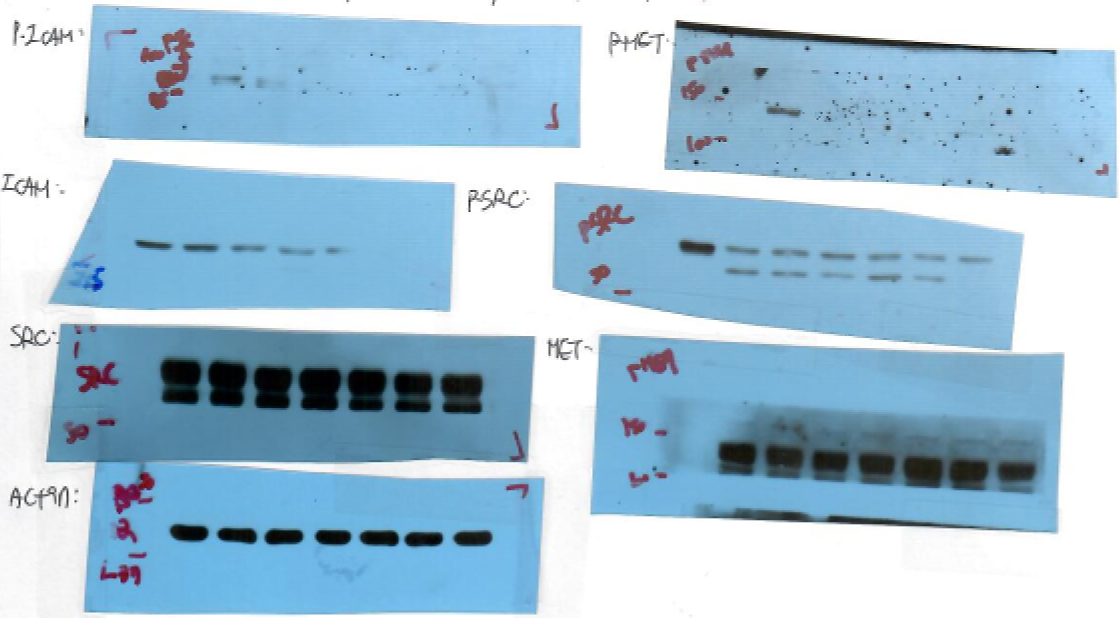

4E SW480

HGF(-): si-C, si-IC

HGF(+): si-C, si-IC

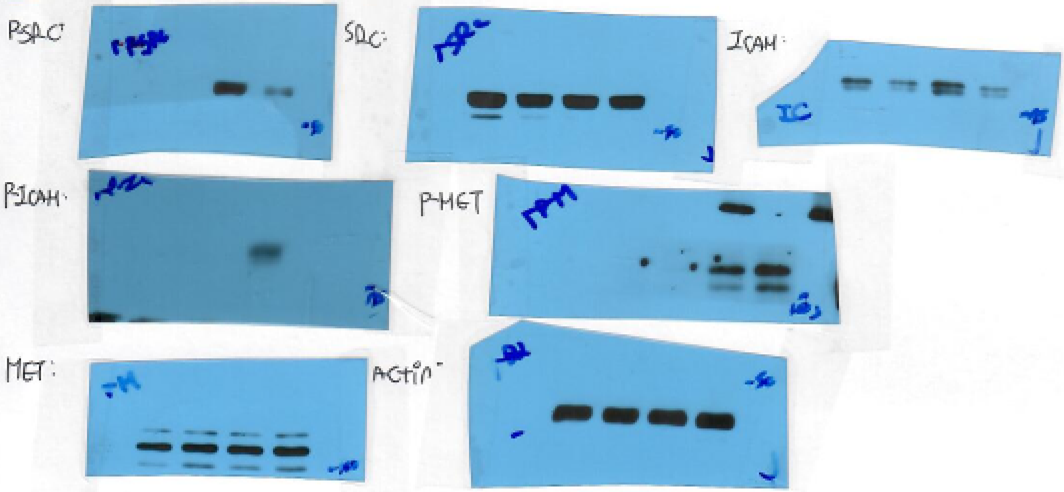

Fig. 4

4F: HT29   C-MET: — — — — + +
ICAM1: — WT YA YD — WT

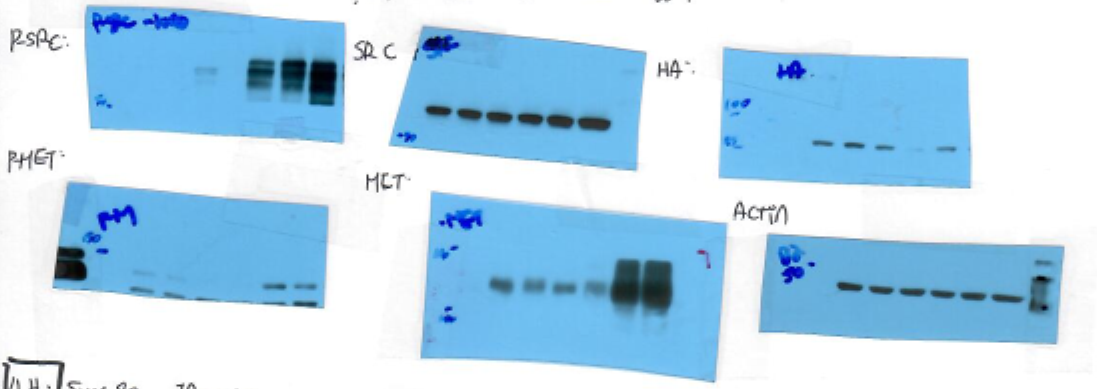

4H: Sw480   IP-ICAM

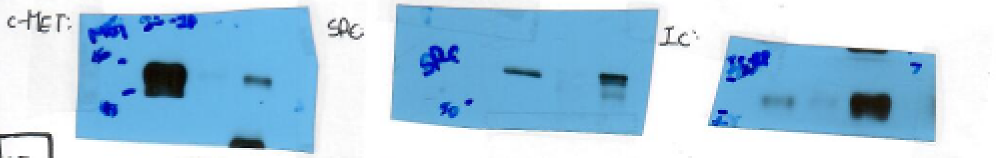

4I: Sw480   Input: si-C si-IC   IgG   IP: si-C si-IC

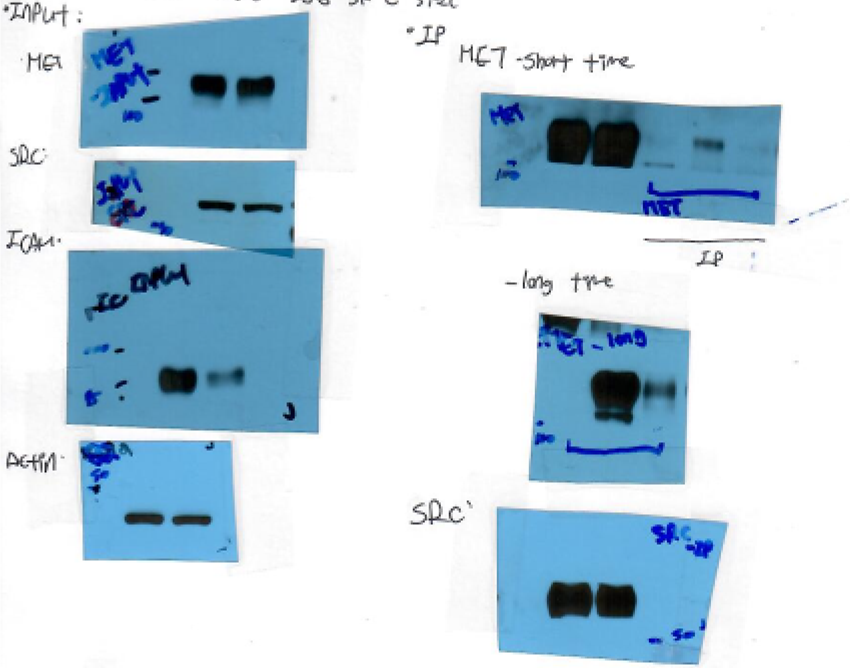

Fig. 4

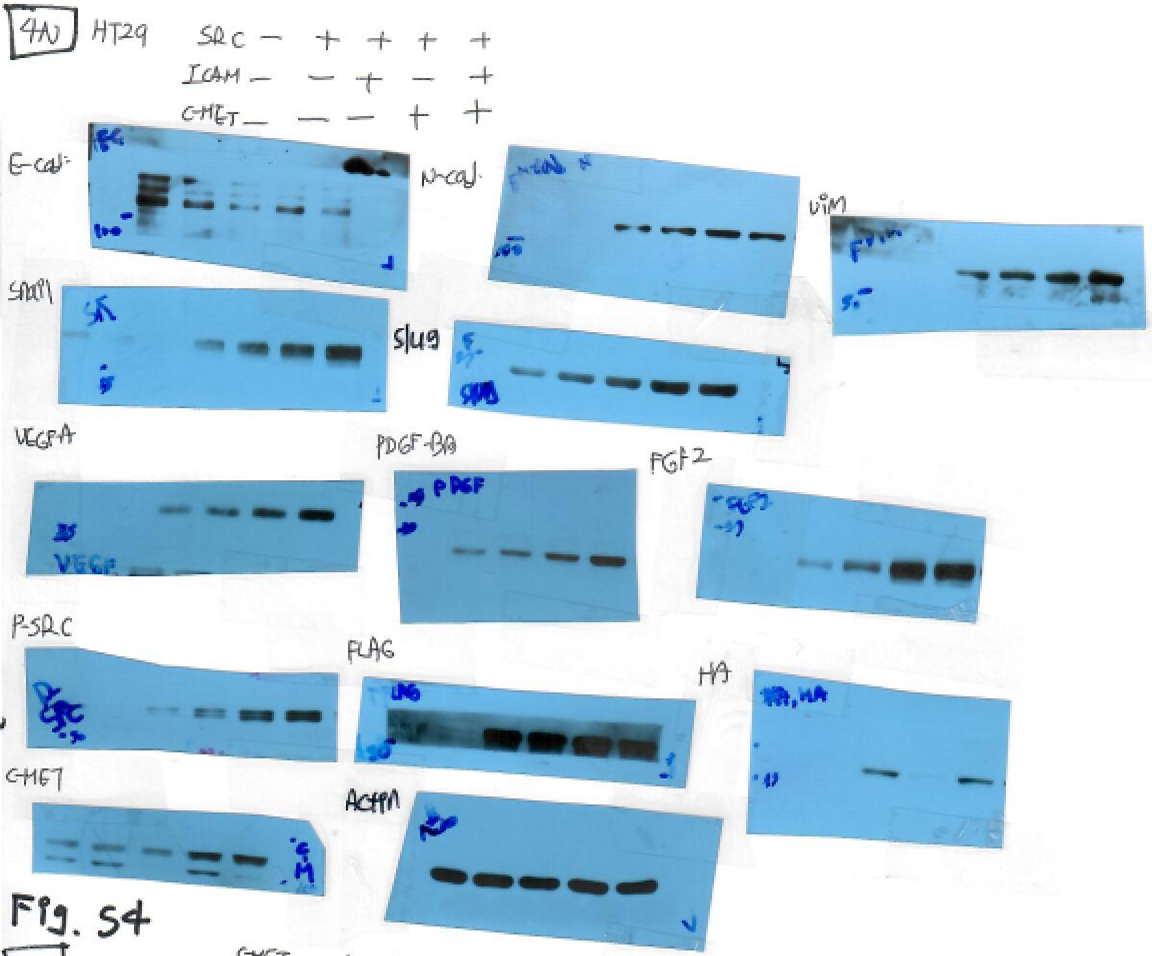
4N
HT29
SRC − + + + +
ICAM − − + − +
C-MET − − − + +
E-cad
N-cad
Vim
SNAIL
Slug
VEGF-A
PDGF-BB
FGF2
P-SRC
FLAG
HA
C-MET
Actin

Fig. S4

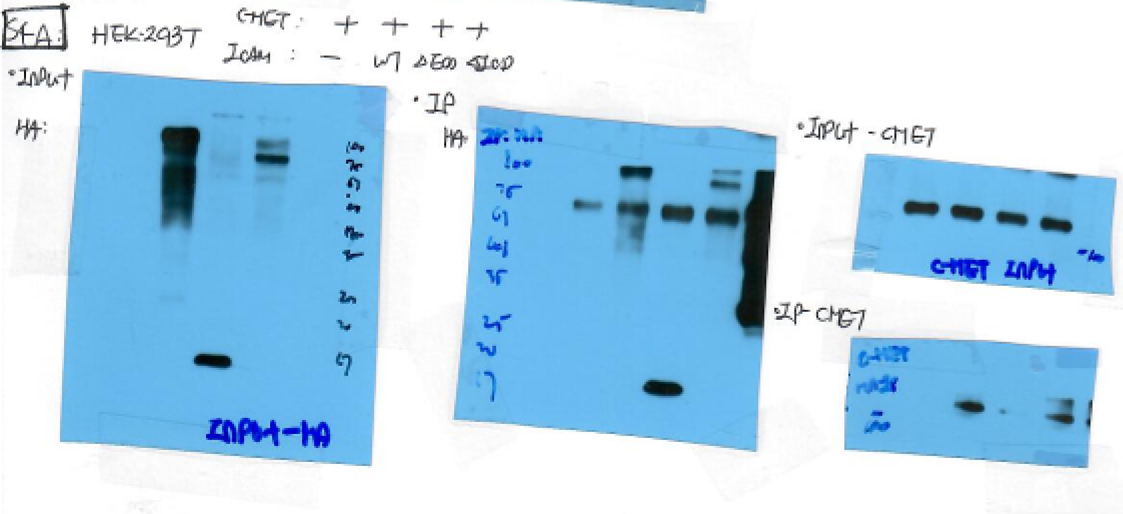
S4A
HEK-293T
C-MET : + + + +
ICAM : − WT ΔE00 Δ1000
• Input
HA
• IP
HA
• Input - C-MET
• IP - C-MET
INPUT-HA

Fig. S4

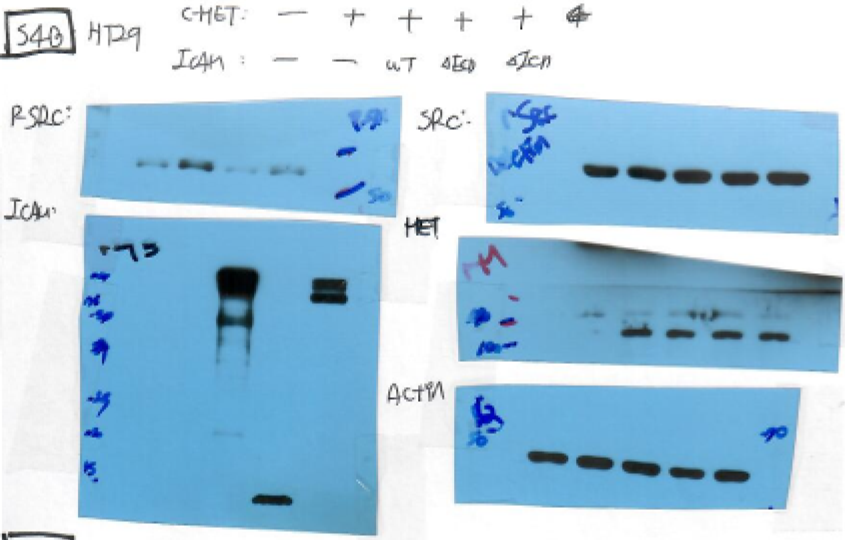
S4B
HT29
C-MET: — + + + +
ICAM: — — WT ΔECD ΔCYT
P-SRC:
SRC:
ICAM:
MET
ACTIN

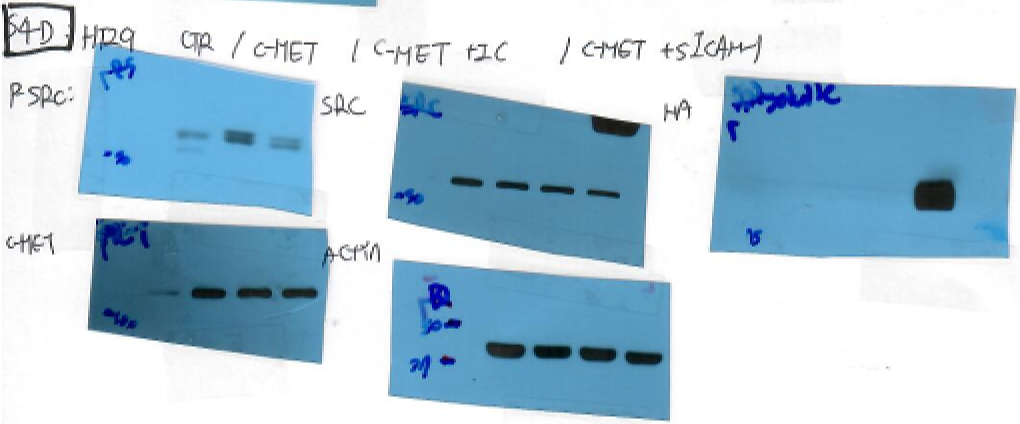
S4D
HT29
CTR / C-MET / C-MET +IC / C-MET +sICAM-1
P-SRC:
SRC
HA
C-MET
ACTIN

Fig. 5

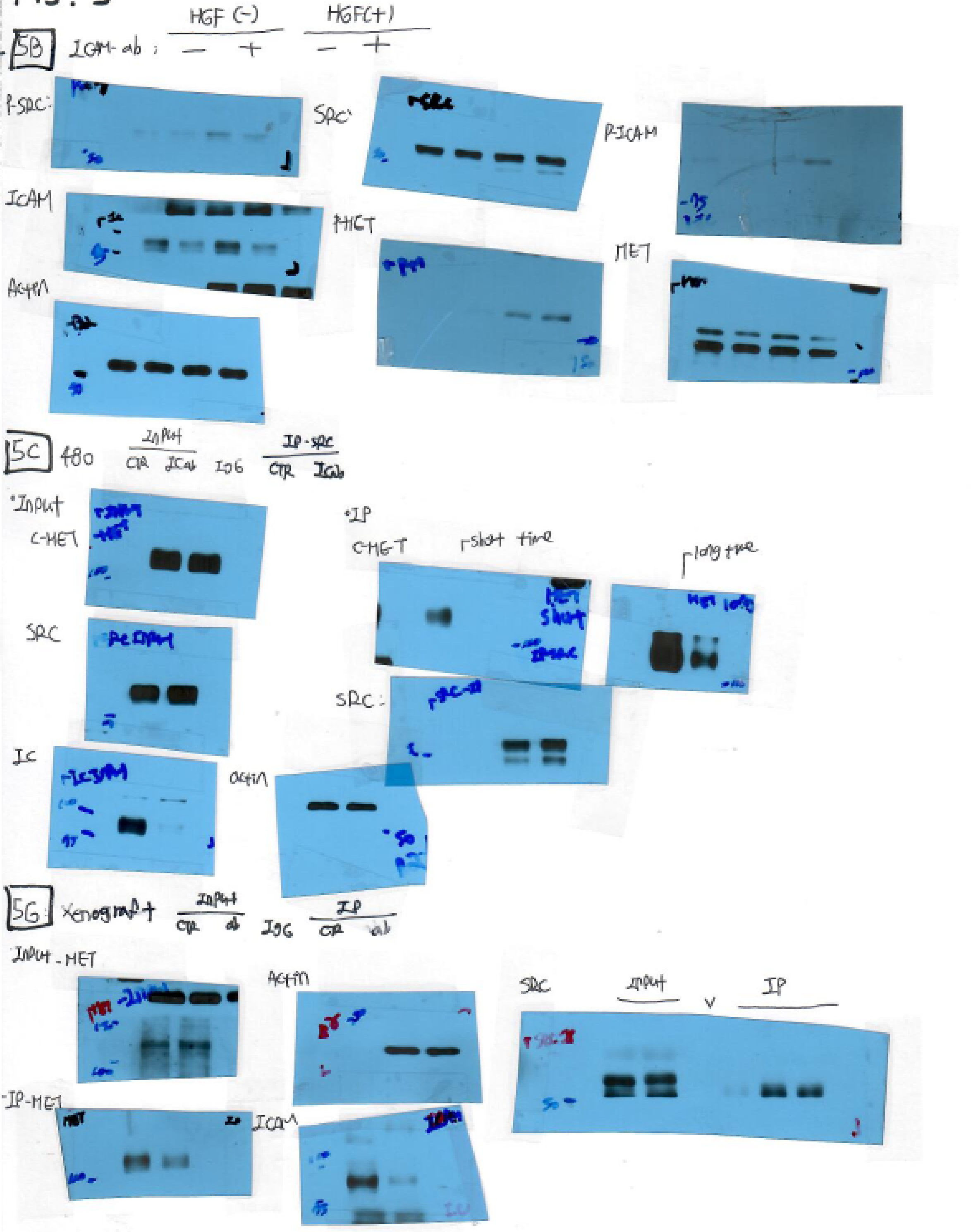
5B
ICAM-ab :
HGF (-)
HGF(+)
− + − +
P-SRC
SRC
P-ICAM
ICAM
P-MET
MET
Actin
5C
480
Input
CTR
ICab
IgG
IP-SRC
CTR
Icab
Input
C-MET
IP
C-MET
short time
long time
SRC
SRC
IC
actin
5G
Xenograft
Input
CTR
ab
IgG
IP
CTR
ab
Input-MET
Actin
SRC
Input
IP
IP-MET
ICAM

Fig_S5

S5A Sw480 : CTR / Icam-ab

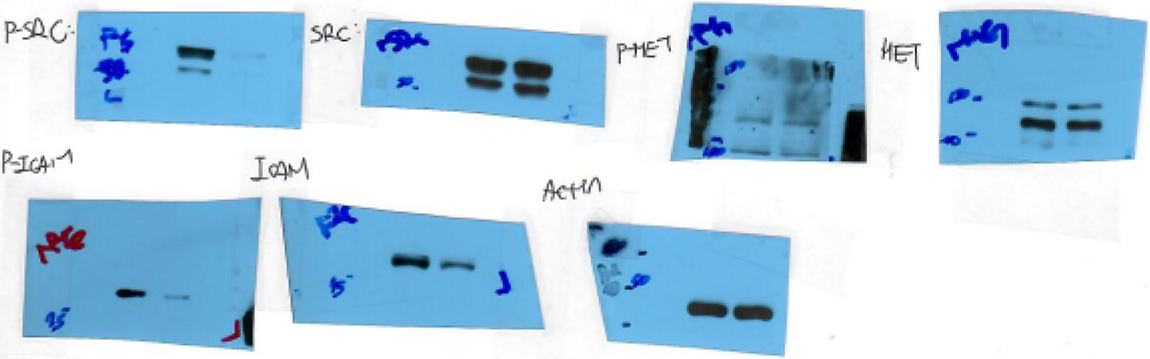

S5C Sw480 : CTR / ICAM-ab

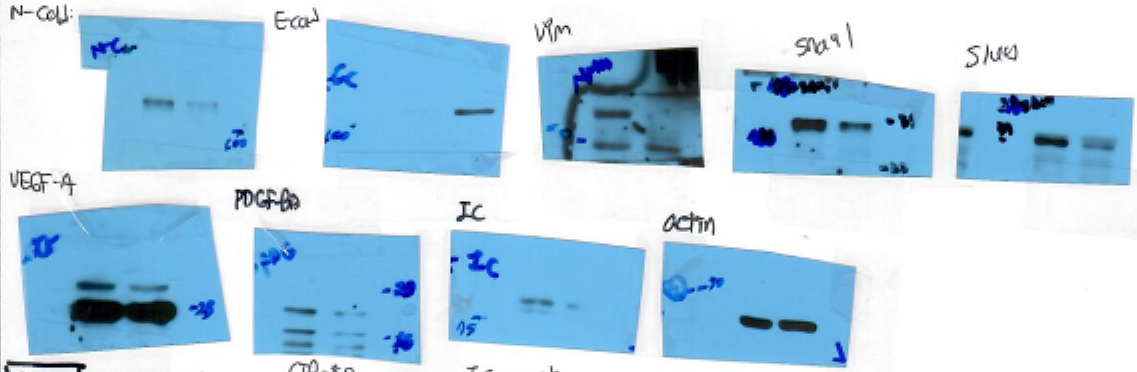

S5D Xenograft CTR-80 #1 #2 #3 ICam-ab #1 #2 #3

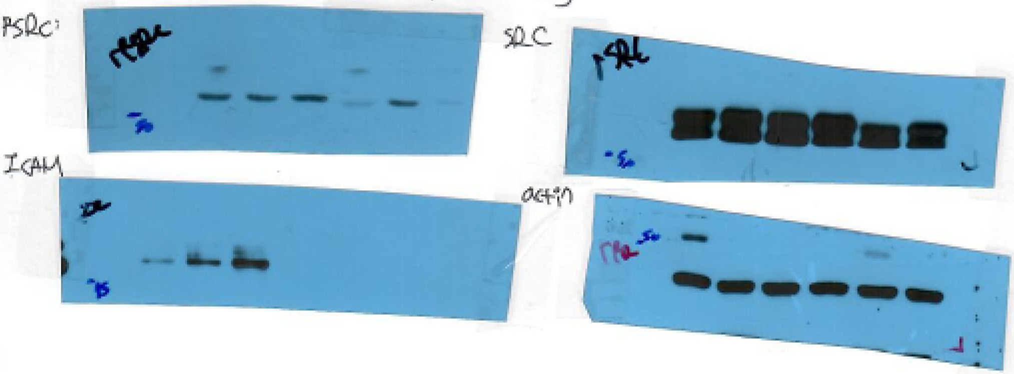

Fig_S5

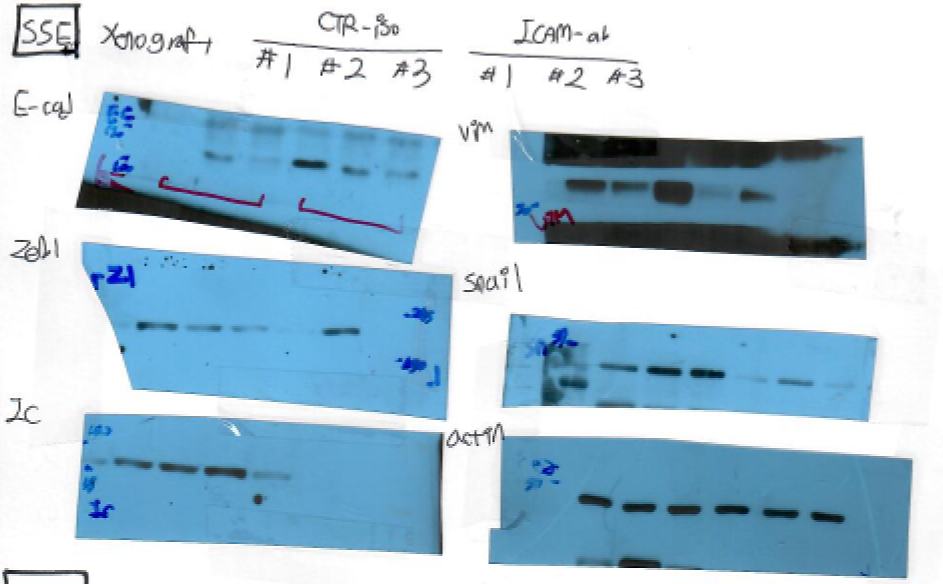
S5E
Xenograft
CTR-iso
#1 #2 #3
ICAM-ab
#1 #2 #3
E-cad
Vim
Zeb1
Snail
ZC
actin

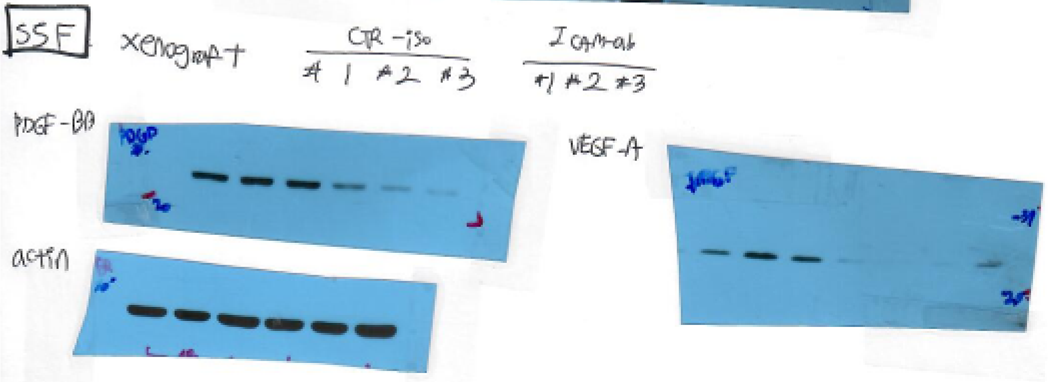
S5F
Xenograft
CTR-iso
#1 #2 #3
ICAM-ab
#1 #2 #3
PDGF-BB
VEGF-A
actin
