# Supplementary material for: ICAM-1 promotes cancer progression by regulating SRC activity as an adapter protein in colorectal cancer
Source: Cell Death Dis. 2022 Apr 29;13(4):417. doi: 10.1038/s41419-022-04862-1 (PMC9054780; doi:10.1038/s41419-022-04862-1)

Fig.1G

* SW-480 : si-CTR / si-ICAM-1 / si-ICAM-1 #2

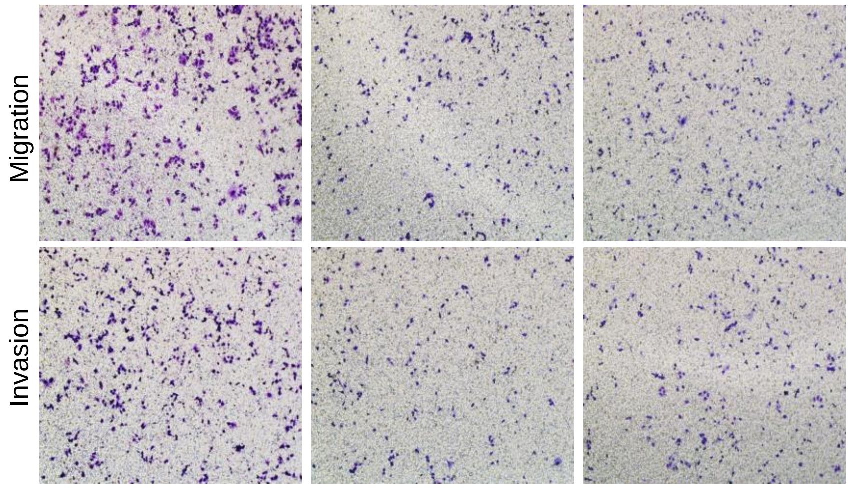

Fig.1I

* Mouse xenograft : sh-CTR / sh-ICAM-1

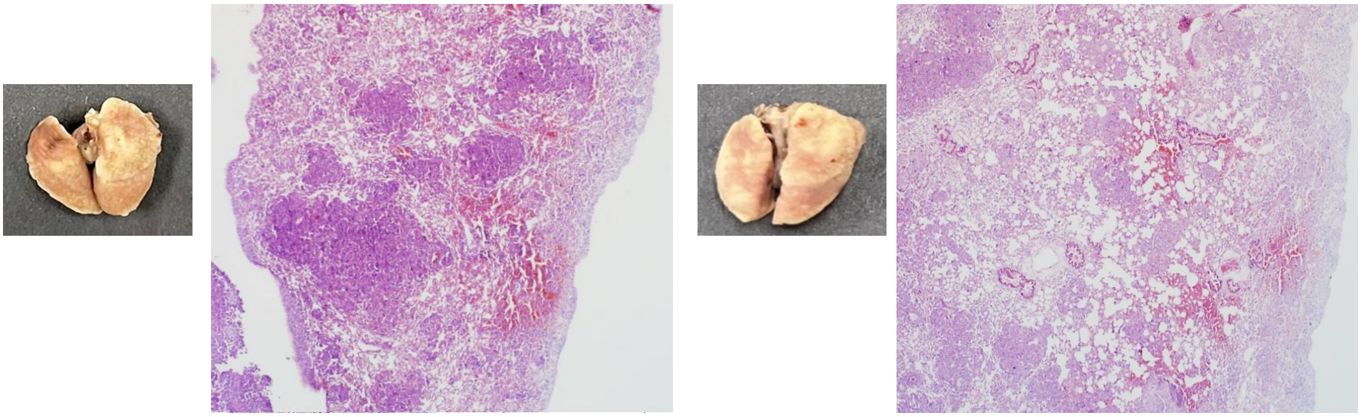

Fig.1K

* Mouse xenograft : sh-CTR / sh-ICAM-1

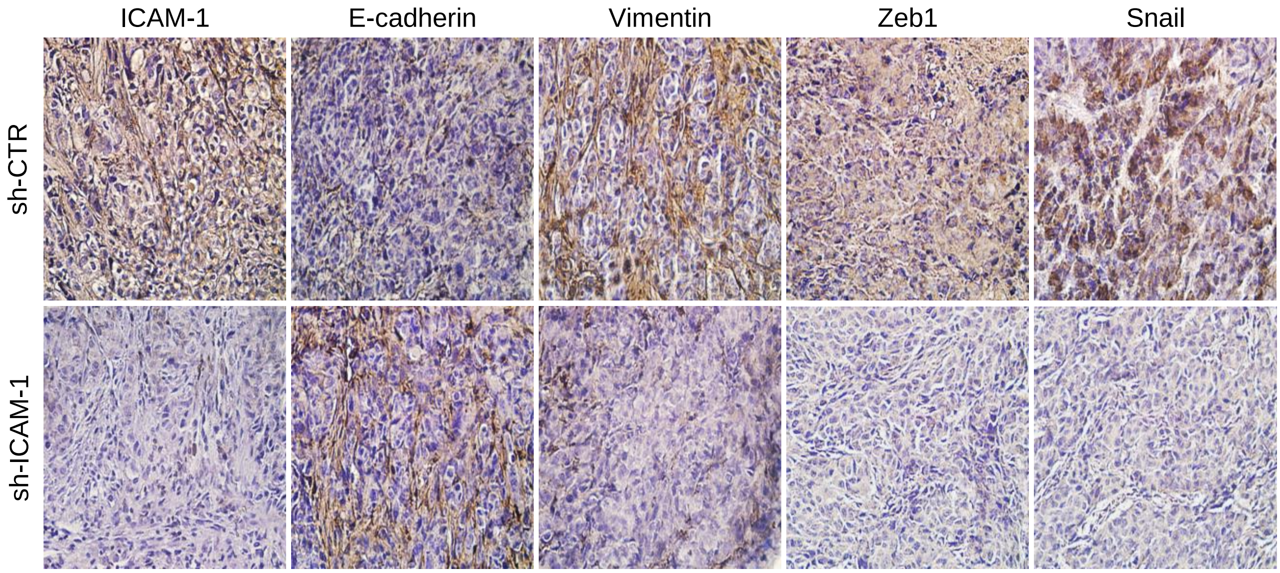

Fig.1L

* SW-480 : si-CTR / si-ICAM-1 / si-ICAM-1 #2

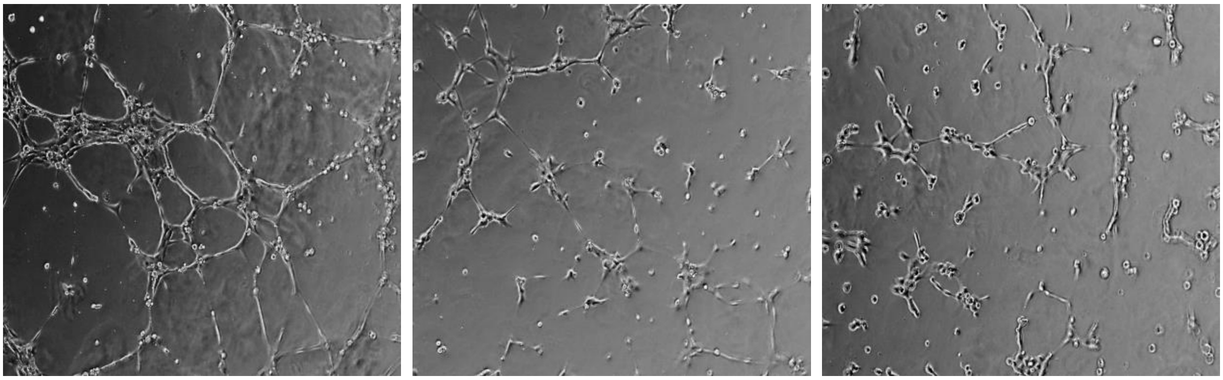

Fig.1N

* Mouse xenograft : sh-CTR / sh-ICAM-1

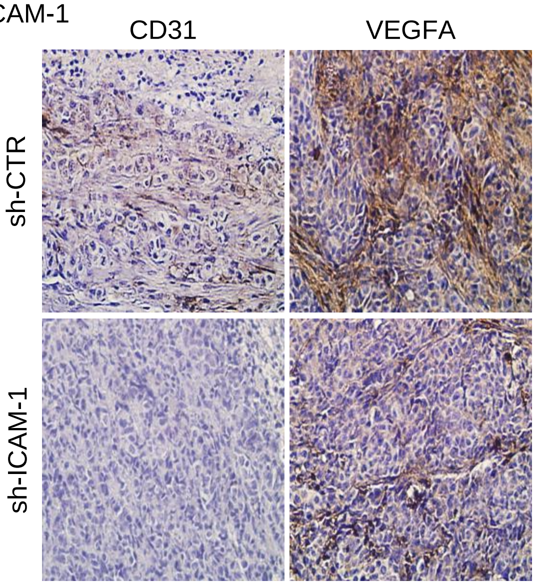

Fig.1O

* Mouse xenograft : sh-CTR / sh-ICAM-1

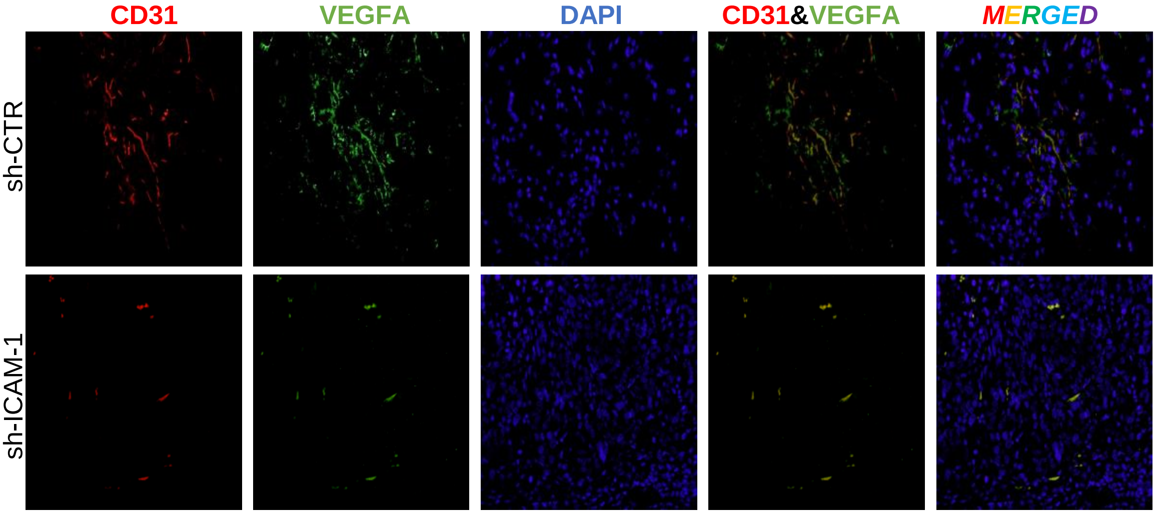

Fig.S1C

* HT-29 : CTR / ICAM-1 O/E

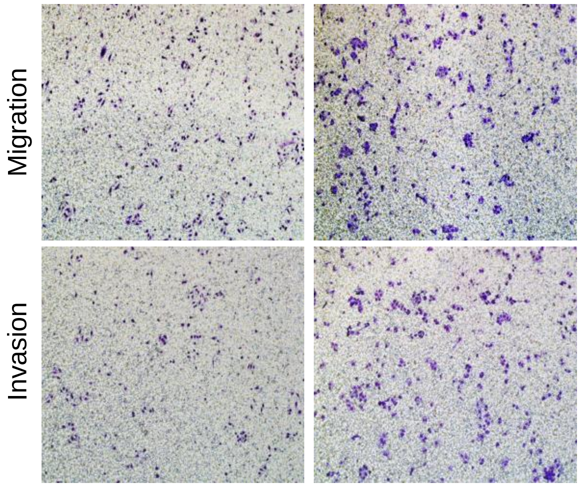

Fig.1D

* HT-29 : CTR / ICAM-1 O/E

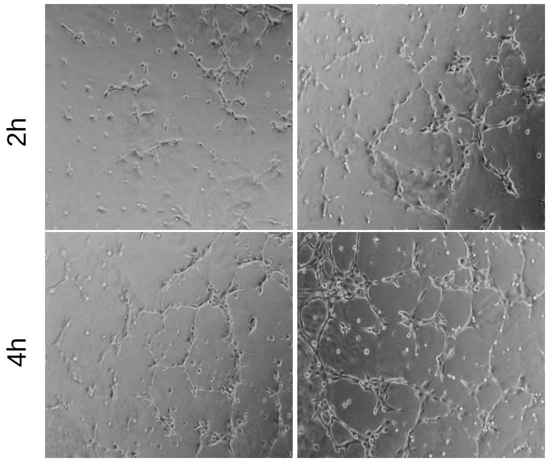

Fig.1H

* Mouse xenograft – Tail vein injection : sh-CTR / sh-ICAM-1

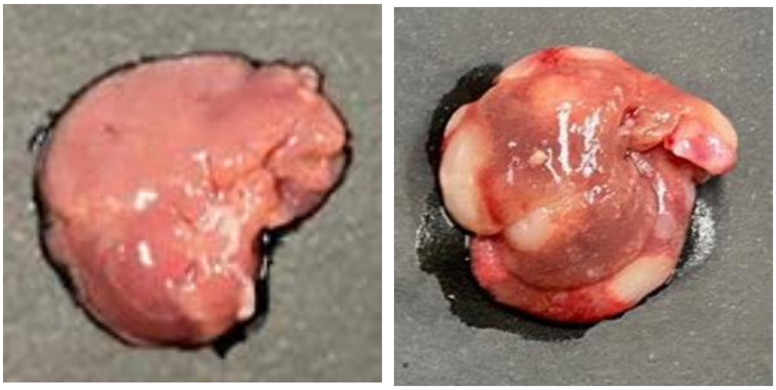

Fig.1I

* Mouse xenograft – Tail vein injection : sh-CTR / sh-ICAM-1

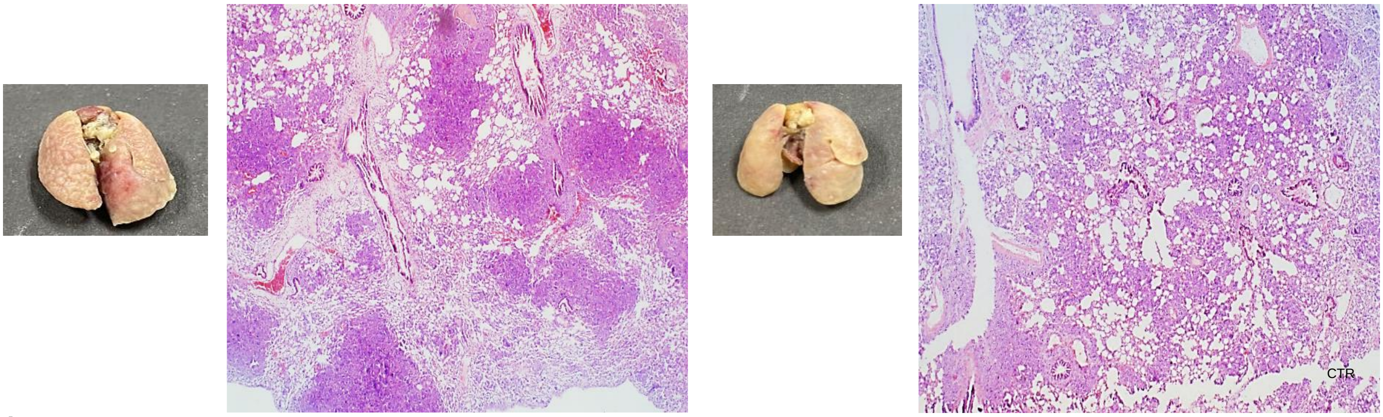

Fig.2F

* Mouse xenograft : sh-CTR / sh-ICAM-1

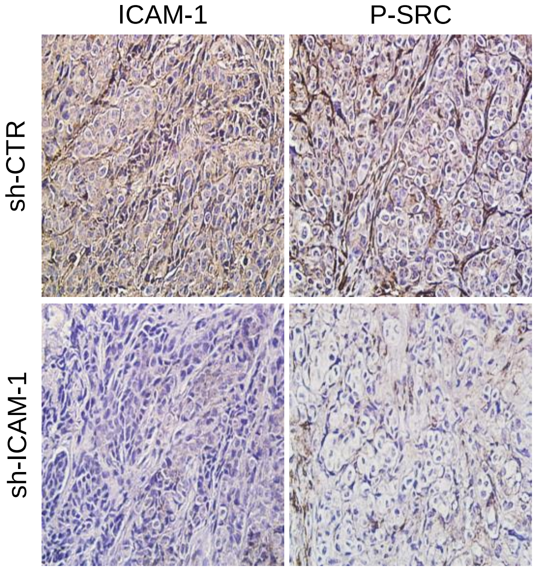

Fig.2H

* HT-29 : CTR / ICAM-1 O/E / ICAM-1 O/E +SRCi

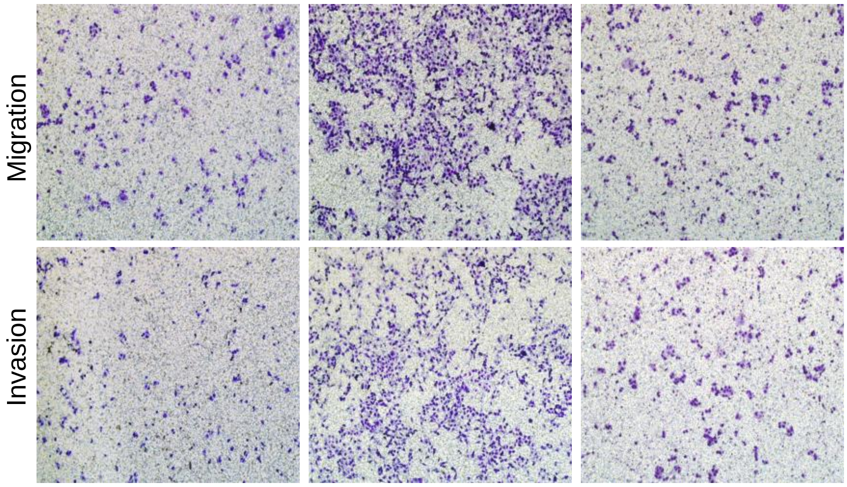

Fig.2I

* HT-29 : CTR / ICAM-1 O/E / ICAM-1 O/E +SRCi

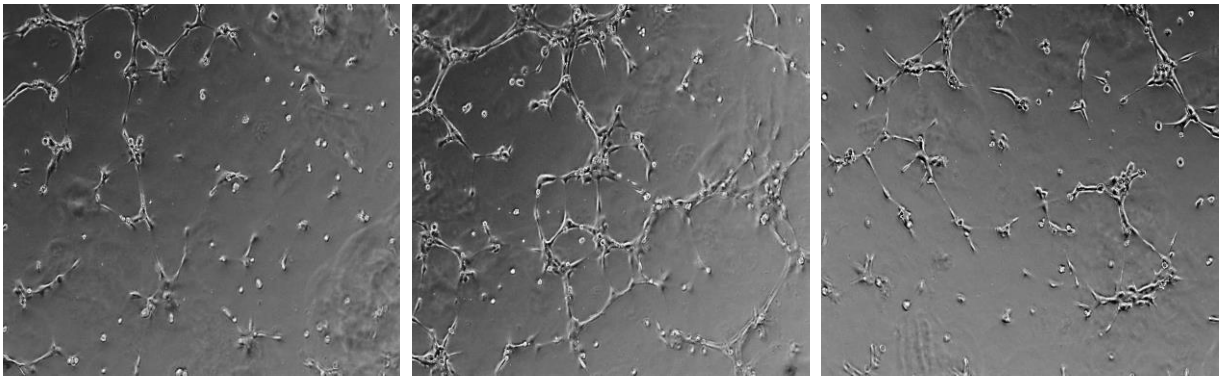

Fig.2L

* HT-29 : CTR / ICAM-1 O/E / ICAM-1 O/E +STAT3i

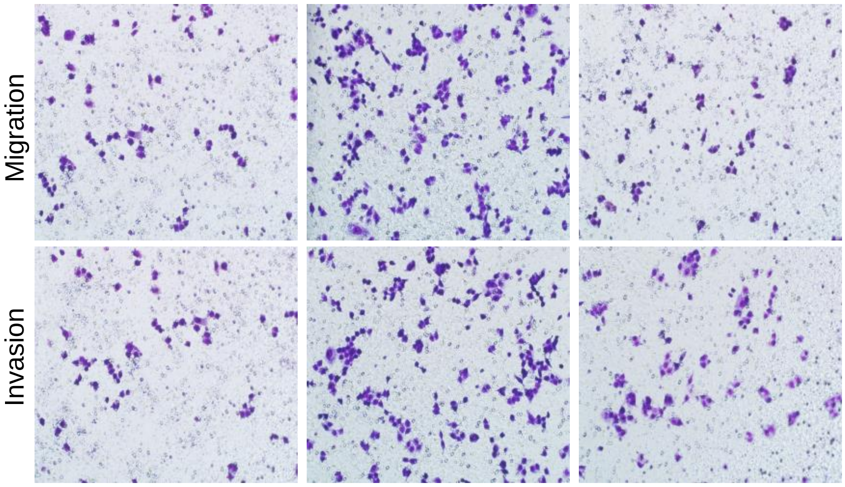

Fig.2M

* HT-29 : CTR / ICAM-1 O/E / ICAM-1 O/E +STAT3i

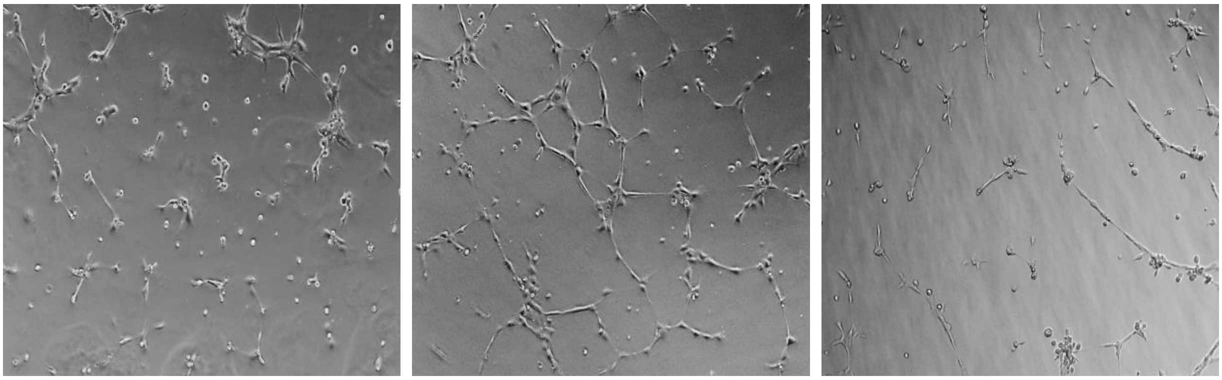

Fig.S2D

* SW-480 : si-CTR / si-SRC #1 / si-SRC #2

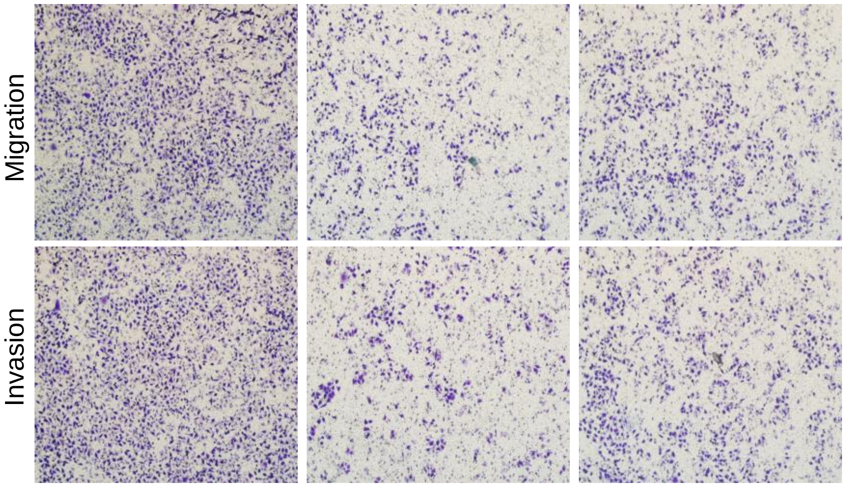

Fig.S2E

* SW-480 : si-CTR / si-SRC #1 / si-SRC #2

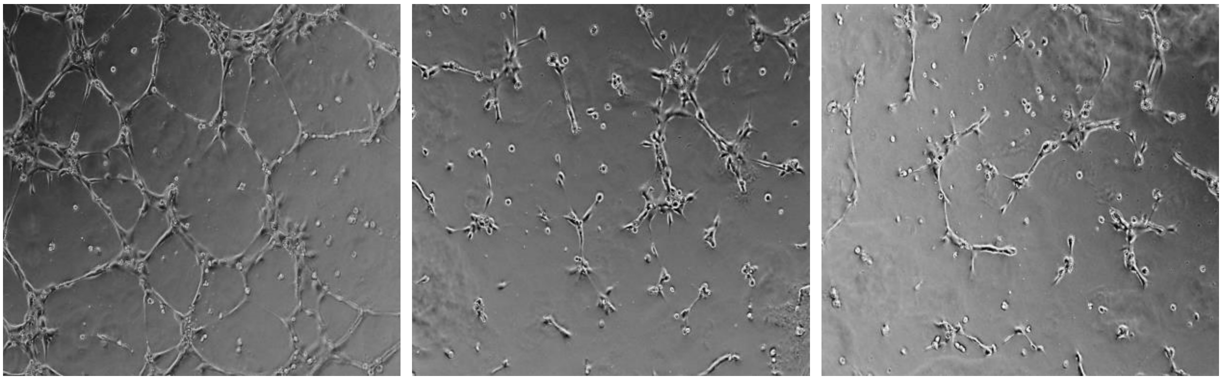

Fig.S2G

* HT-29 : CTR / SRC O/E

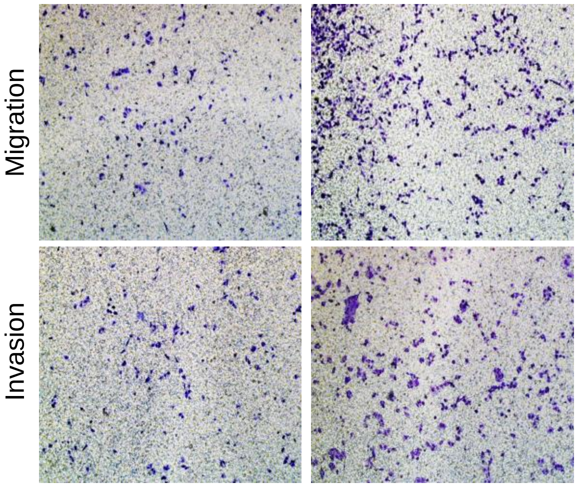

Fig.S2H

* HT-29 : CTR / SRC O/E

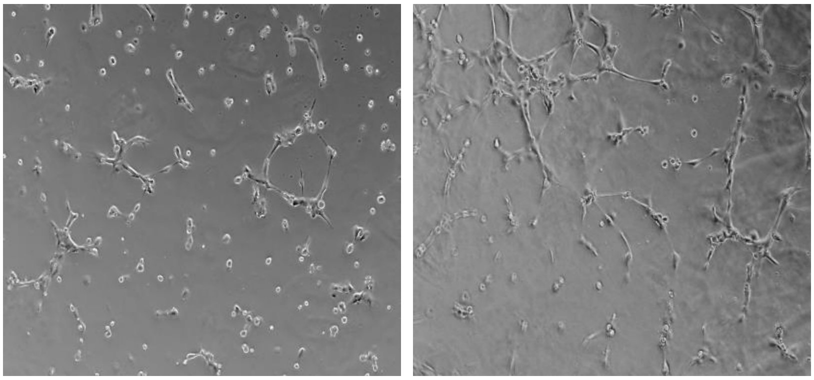

Fig.S2J

* HT-29 : CTR / ICAM-1 / ICAM-1 +si-SRC #1 / ICAM-1 +si-SRC #2

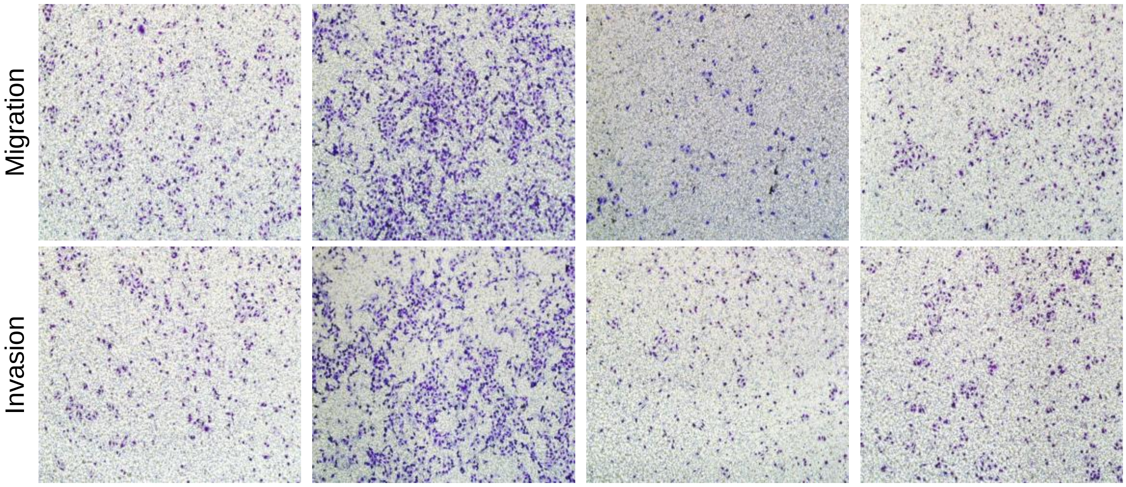

Fig.S2K

* HT-29 : CTR / ICAM-1 / ICAM-1 +si-SRC #1 / ICAM-1 +si-SRC #2

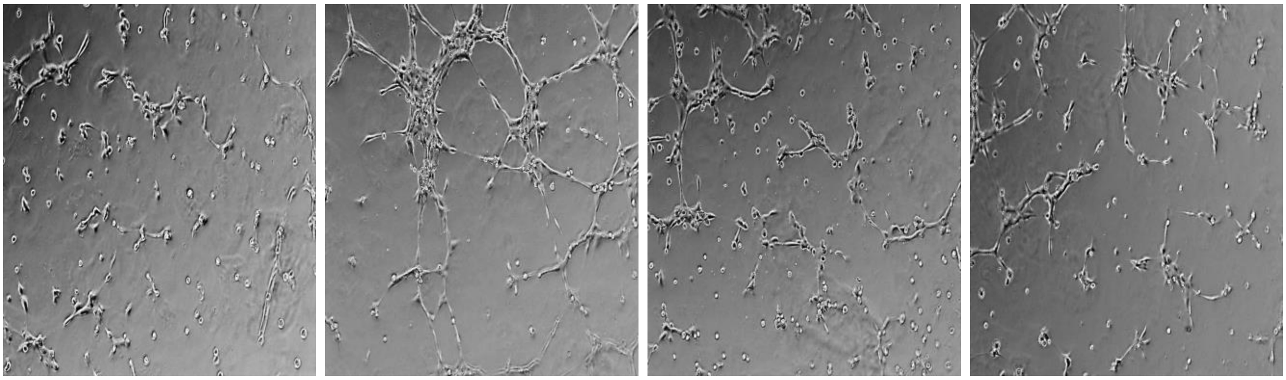

Fig.3B – PLA

* SW-480 : ICAM-1 / ICAM1 +SRC

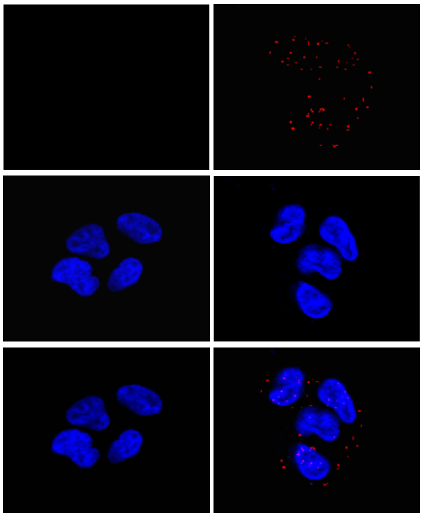

Fig.3G - PLA

* 293T : - / WT / YA / YD

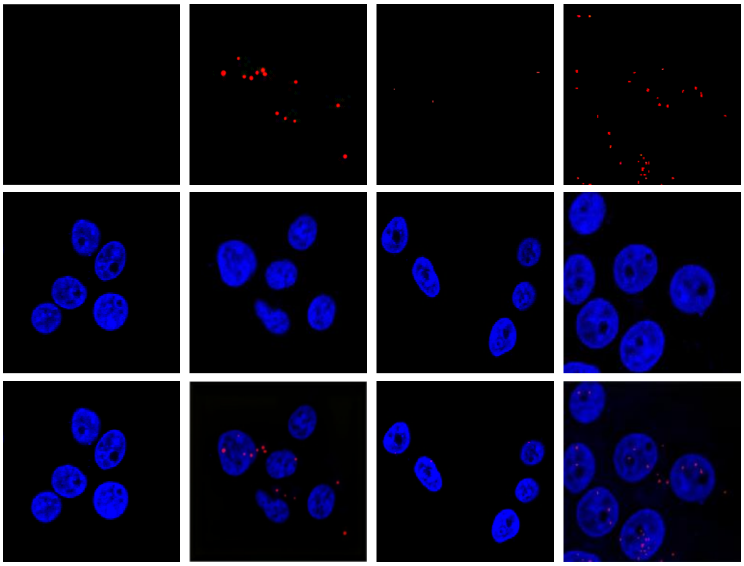

Fig.3K

* HT-29 : - / WT / YA / YD

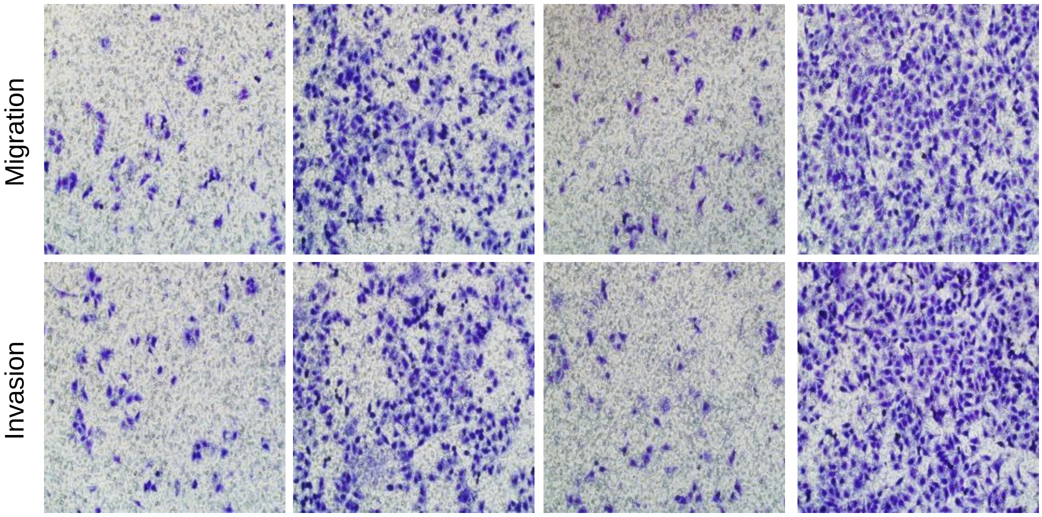

Fig.3M

* HT-29 : - / WT / YA / YD

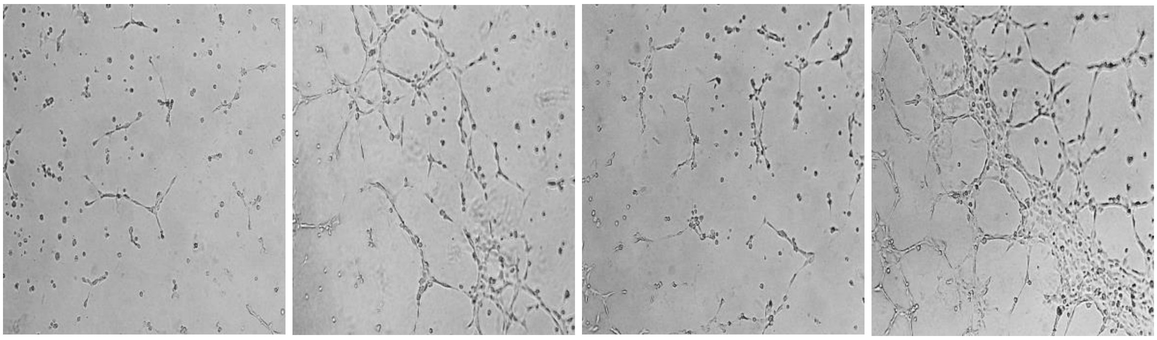

Fig.S3A -PLA

* 293T : - / WT / ΔECD / ΔICD

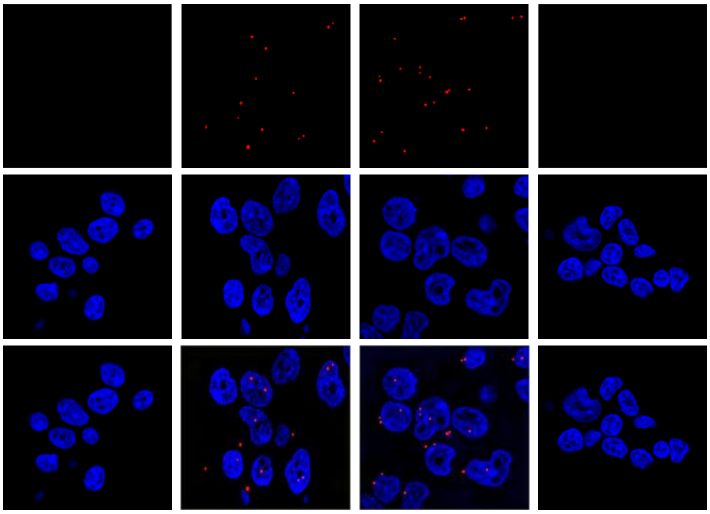

Fig.4J - PLA

* SW-480 : CTR / si-ICAM-1

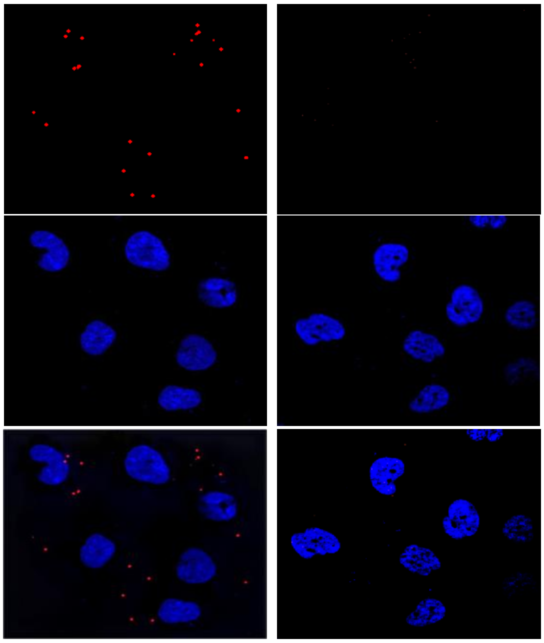

Fig.4K - ICC

* HT-29 : SRC / SRC+IC / SRC+MET / SRC+IC+MET

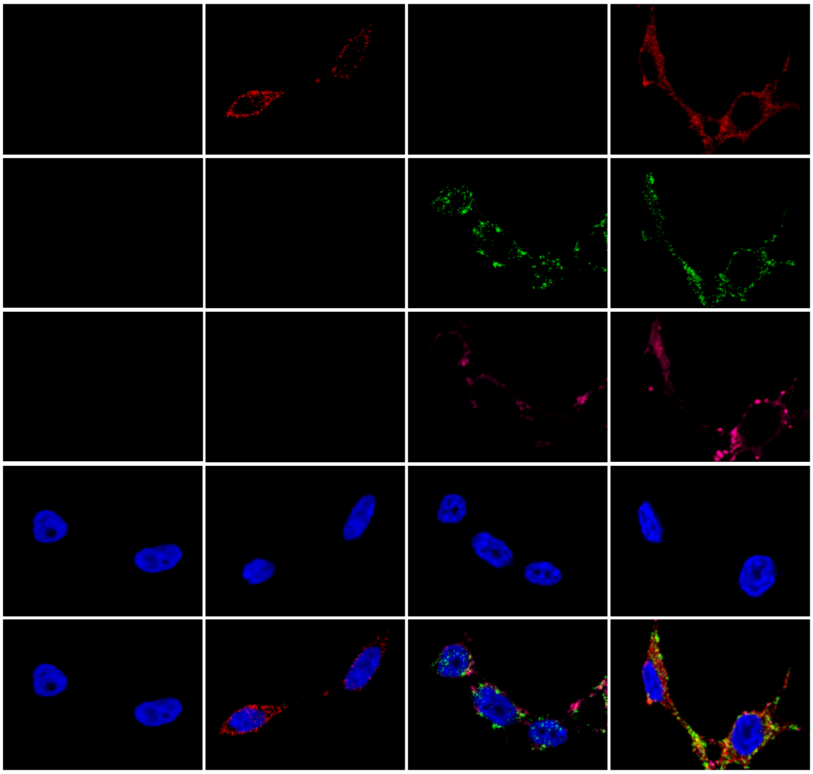

Fig.4L

* HT-29 : SRC / SRC+IC / SRC+MET / SRC+IC+MET

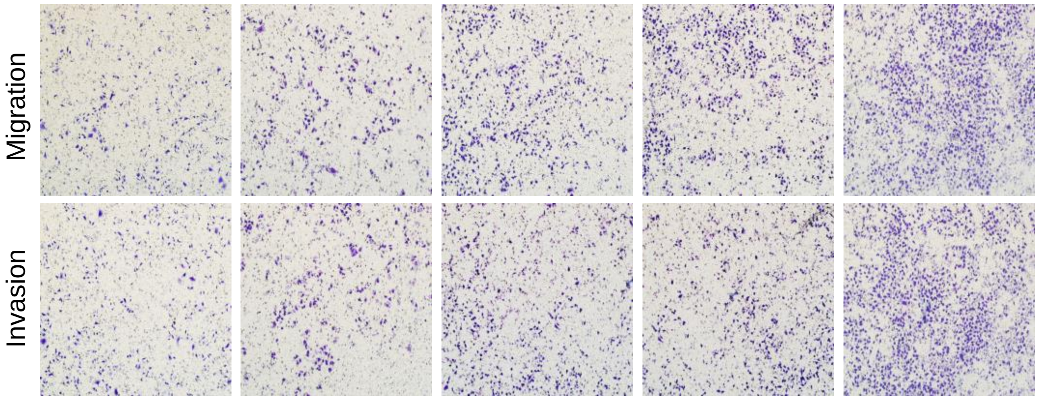

Fig.4M

* HT-29 : SRC / SRC+IC / SRC+MET / SRC+IC+MET

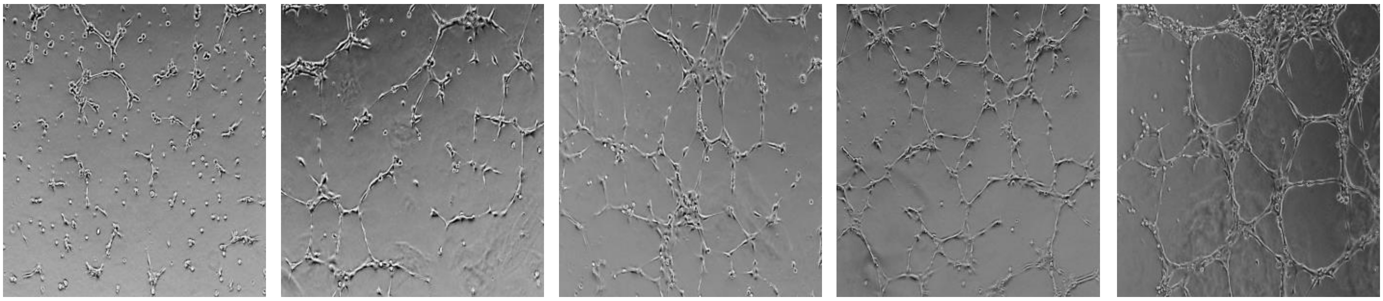

Fig.5D

* SW-480 : CTR / ICAM-1 ab

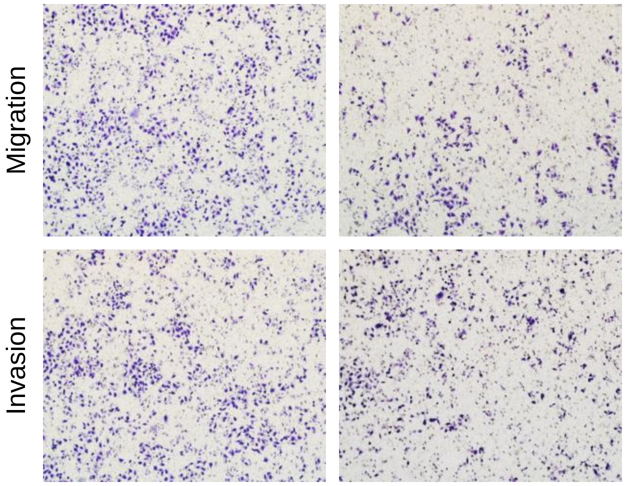

Fig.5E

* SW-480 : CTR / ICAM-1 ab

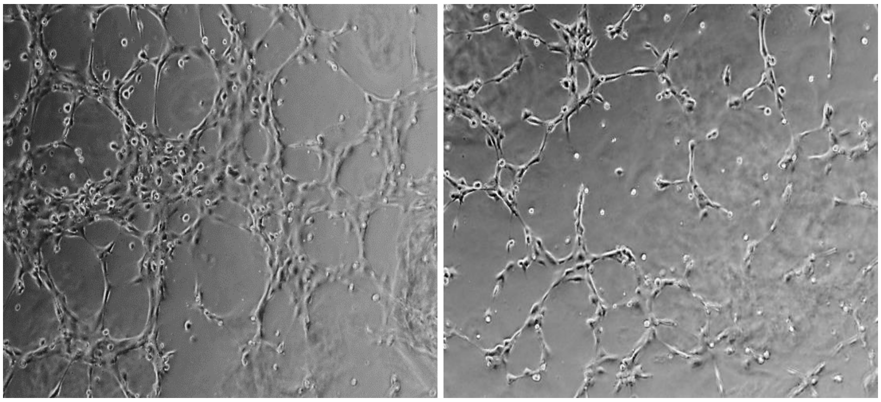

Fig.5H

* Xenograft : CTR / ICAM-1 ab

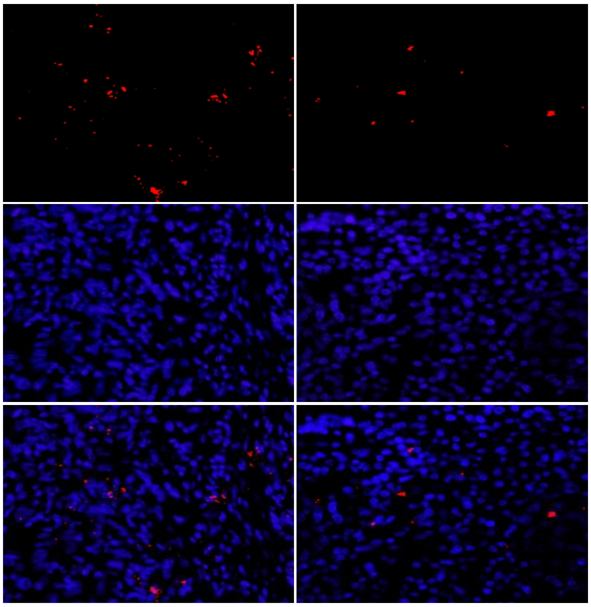

Fig.5I

* Xenograft : CTR / ICAM-1 ab

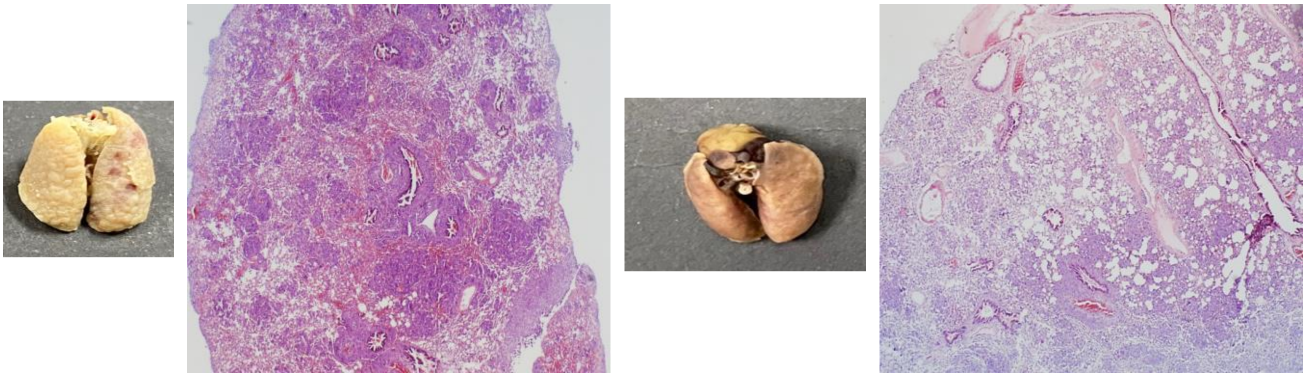

Fig.5K

* Xenograft : CTR / ICAM-1 ab

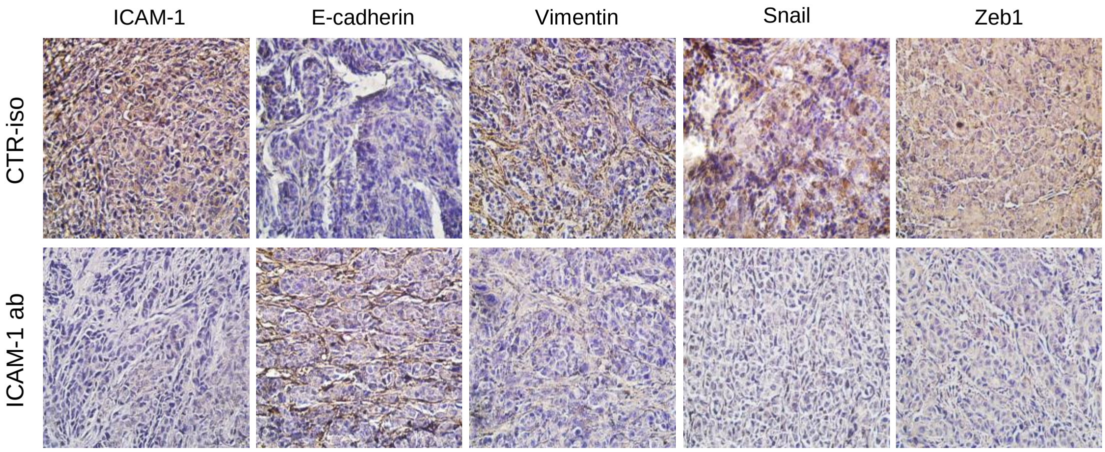

Fig.5N

* Xenograft : CTR / ICAM-1 ab

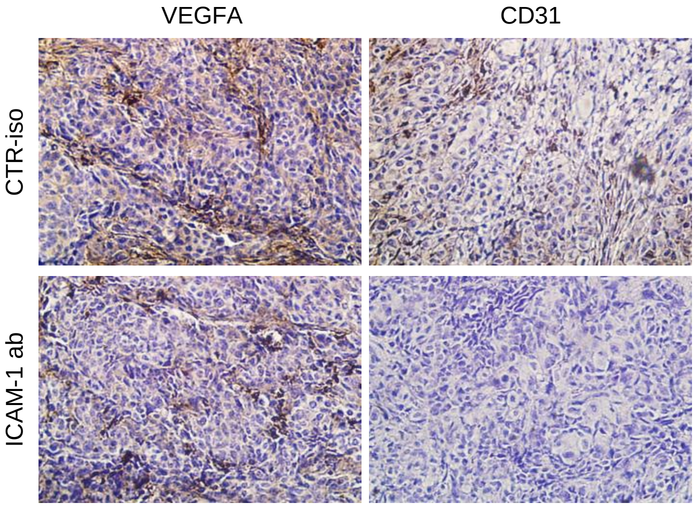

Fig.5P

* Xenograft : CTR / ICAM-1 ab

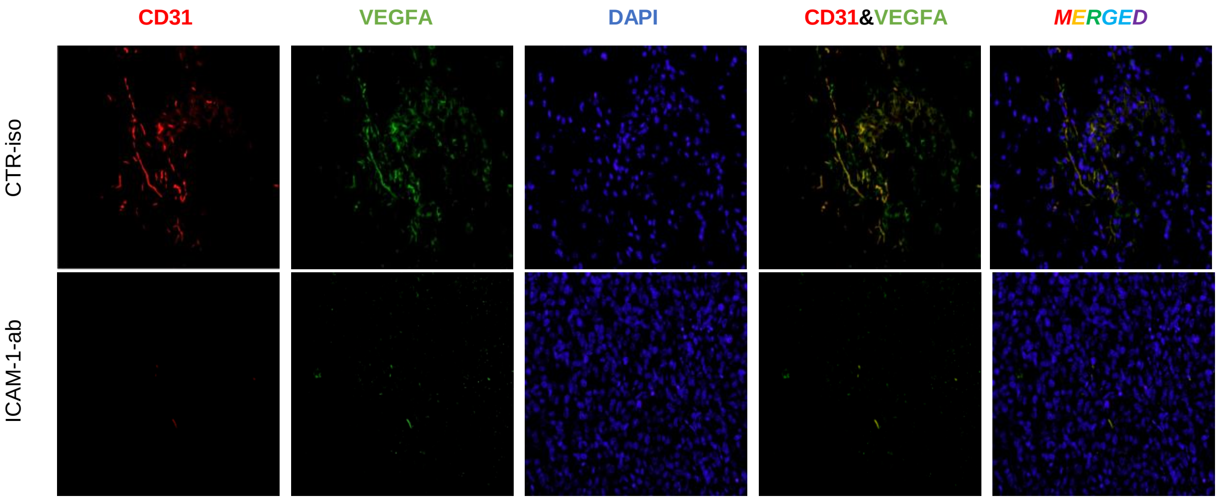

Fig.6B

* Patient tissue array – ICAM-1 staining :stage 1 /2 /3 /4

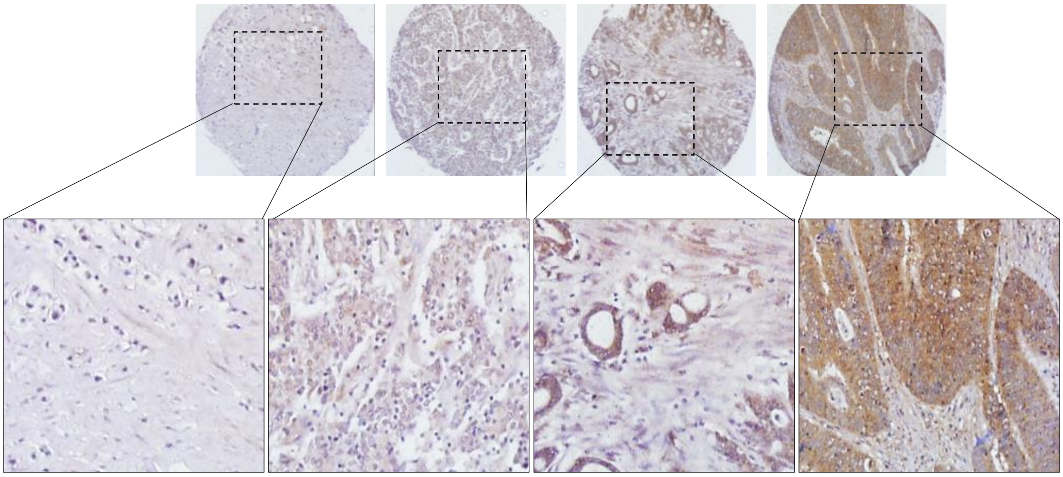

Fig.6E

* Patient tissue array – ICAM-1 & p-SRC staining

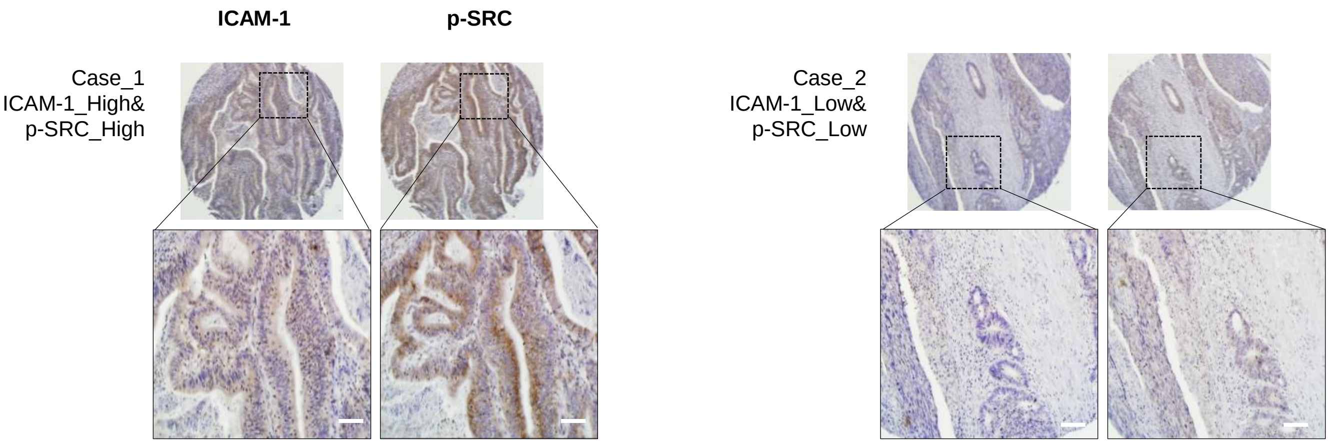

Supplement: Supplementary file 3 — raw data_staining [file 41419_2022_4862_MOESM3_ESM.pdf]
